# Novel Solid Forms of Cardarine/GW501516 and Their Characterization by X-Ray Diffraction, Thermal, Computational, FTIR, and UV Analysis

**DOI:** 10.3390/pharmaceutics17020152

**Published:** 2025-01-23

**Authors:** Alexandru Turza, Maria Bosca, Marieta Muresan-Pop, Liviu Mare, Gheorghe Borodi, Violeta Popescu

**Affiliations:** 1 National Institute For R&D of Isotopic and Molecular Technologies, 67-103 Donat, 400293 Cluj-Napoca, Romania; alexandru.turza@itim-cj.ro; 2 Department of Physics and Chemistry, Technical University of Cluj-Napoca, 400114 Cluj-Napoca, Romania; maria.bosca@phys.utcluj.ro (M.B.); liviu.mare@campus.utcluj.ro (L.M.); violeta.popescu@chem.utcluj.ro (V.P.); 3 Institute of Interdisciplinary Research in Bio-Nano-Sciences, Babes Bolyai University, 42 Treboniu Laurian, 400271 Cluj-Napoca, Romania; marieta.muresan@ubbcluj.ro

**Keywords:** GW501516, cardarine, polymorphism, crystal structure, cocrystal, solubility

## Abstract

Cardarine (C_21_H_18_F_3_NO_3_S_2_), better known by the popular name of GW501516, is a peroxisome proliferator-activated receptor delta (PPR-δ) agonist that presents potential use in the approach of cardiovascular diseases and metabolic disorders, dyslipidemia, and insulin resistance. The capability of cardarine to exhibit new solid forms by recrystallization from a broad class of solvents was explored. A total of four new solid forms were obtained: a new polymorph of cardarine (C_21_H_18_F_3_NO_3_S_2_), the cardarine: 4,4′-bipyridine cocrystal (C_21_H_18_F_3_NO_3_S_2_·0.5C_10_H_8_N_2_), the cardarine methanol solvate (C_21_H_18_F_3_NO_3_S_2_·CH_3_OH), and the cardarine dimethylformamide solvate (C_21_H_18_F_3_NO_3_S_2_·C_3_H_7_NO). Moreover, two derivatives of cardarine were obtained, in the form of the mono-oxidized cardarine structure (C_21_H_18_F_3_NO_4_S_2_) and the dioxidized cardarine structure (C_21_H_18_F_3_NO_5_S_2_). The formation process was proven by the determination of their crystal structures using single crystal X-ray diffraction and followed by their lattice energies evaluation. Further investigations have been conducted by powder X-ray diffraction, DTA/TGA thermal analysis, and FTIR spectroscopy. The stability and solubility were analyzed as well.

## 1. Introduction

Cardarine, also known by the name of GW501516 (2-[2-methyl-4-[[4-methyl-2-[4-(trifluoromethyl)phenyl]-1,3-thiazol-5-yl]methylsulfanyl]phenoxy]acetic acid), is a peroxisome proliferator-activated receptor delta (PPAR-δ) agonist [1] developed in the 1990s as a compound with potential use in cardiovascular and metabolic disorder treatment. Previous studies have shown that cardarine is efficient and has ameliorated some obesity-related parameters such as oxidative stress dyslipidemia and insulin resistance, which are related to metabolic disorders. The PPAR-δ agents developed, including cardarine, are increasing the body’s capacity to use fat as a primary source of fuel as an alternative to glucose [2,3]. The boost in fatty acid metabolism is gained based on the PPAR-δ receptor activation by the upregulation of fatty acid uptake and oxidation [4,5]. Recent studies demonstrated that cardarine is efficient in the treatment of insulin resistance [6] and subepithelial fibrosis during asthma as well [7]. Due to the fact that it boosts the body’s cardiovascular output, it gained rapid popularity among athletes and recreational users as a performance-enhancement drug [8,9]; nevertheless, its usage is prohibited by the World Anti-Doping Agency [10].

The actual paper aims toward the capabilities of cardarine to be grown as new solid forms, which include cocrystals, salts, polymorphs, and solvates; even though the literature provides an extensive study of cardarine polymorphism [11], the CSD database so far reports only five polymorphs of cardarine [12]. The polymorphism of active pharmaceutical ingredients represents their ability to be packed within the crystal structure in various patterns [13,14,15].

**Scheme 1:**
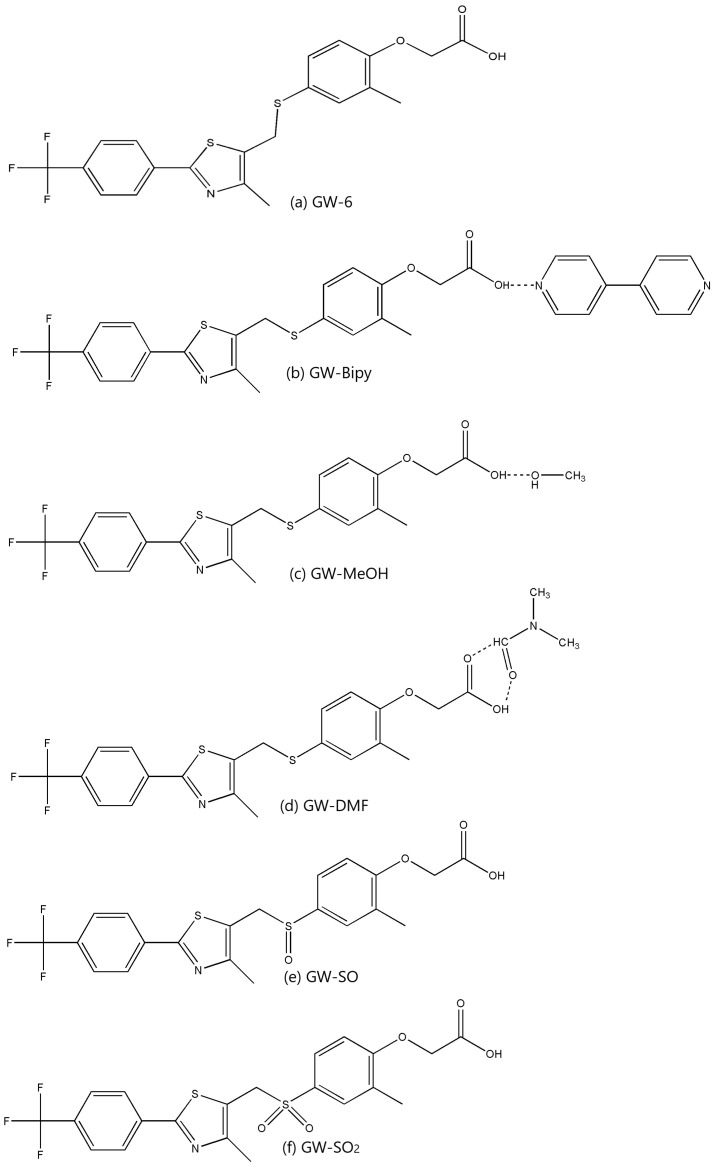
Cardarine solid forms and derivatives.

The formation of solid crystalline forms and multi-component molecular structures that contain at least two constituents are driven by noncovalent contacts such as hydrogen bonds, π···π stacking, and other van der Waals interactions [16,17]. Among the advantages and benefits of the solid forms, the following characteristics are worth mentioning: greater solubility, bioavailability, dissolution rate, melting point, and stability [18,19,20]. The fact that the structural makeup of cardarine, with the carboxyl COOH and the thiazol ring, makes it a suitable candidate for the formation of new crystalline forms via the interactions in a solid by hydrogen bonding must be taken into consideration (Scheme 1).

The recrystallization of cardarine in various solvents was achieved, generating four new solid crystalline forms, which consist of a new polymorph, one 4,4′-bipyridine cocrystal (found in a 2:1 stoichiometric ratio), one methanol solvate, one dimethylformamide solvate, and two new cardarine derivatives, respectively. Their crystal structures were elucidated by single-crystal X-ray diffraction and the supramolecular features were explored by computational methods. Other methods to analyze the materials were DTA/TGA thermal analysis and FTIR spectroscopy. From a pharmaceutical point of view, the solubility of the polymorph, the cocrystal, the methanol, and dimethylformamide solvates were assessed. The stability of the polymorph and the 4,4′-bipyridine cocrystal was evaluated under specific temperature and relative humidity conditions.

## 2. Materials and Methods

### 2.1. Recrystallization and Crystal Growth Experiments

The crystalline powder of cardarine was received from Wuhan Shu Mai Technology Co. (Wuhan, China) and solvents were received from Merck and Sigma-Aldrich (Taufkirchen, Germany).

The new solid forms and their suitable single crystals were prepared by slow evaporation from a wide class of solvents and mixtures. The solutions were kept in the refrigerator at a temperature of 4 °C and evaporated slowly during a period of up to two months. The four prepared crystalline forms and two derivatives are listed below and illustrated in Scheme 1a–f, as follows:

(i) Cardarine polymorph (GW-6, Scheme 1a) was obtained from a solution of methanol;
(ii) Cardarine 4,4′-bipyridine cocrystal (GW-Bipy, Scheme 1b) was obtained from multiple solutions such as acetonitrile, ethanol, and methanol;
(iii) Cardarine methanol solvate (GW-MeOH, Scheme 1c) was prepared in a solution of methanol and water in a 1:1 volumetric ratio;
(iv) Cardarine dimethylformamide solvate (GW-DMF, Scheme 1d) was obtained from a solution of dimethylformamide;
(v) Cardarine, in the mono-oxidized form (GW-SO, Scheme 1e), was obtained from a solution of dimethyl sulfoxide but was not further reproduced;
(vi) Cardarine, in the dioxidized form (GW-SO_2_, Scheme 1f), was obtained from a mixture with nitromethane and was not further reproduced anymore.

### 2.2. Single-Crystal X-Ray Diffraction and Refinement

The single crystals were attached on a fine nylon loop coated by inert oil and mounted on the goniometer of a SuperNova diffractometer equipped with dual X-ray micro-sources (Mo and Cu), which operates at 50 kV and 0.8 mA and Eos CCD detector. The collection of experimental data and their correction process for Lorentz, polarization, and adsorption effects were accomplished in the CrysAlis PRO program [21].

The crystal structures of GW-6 and GW-Bipy were solved by Direct Methods with the SHELXS solution program [22], while GW-MeOH, GW-DMF, GW-SO, and GW-SO_2_ were solved using Intrinsic Phasing with SHELXT [23]. The structures were refined using Least Squares minimization by the SHELXL [24] refinement package. All programs are implemented in the Olex2 package [25].

Hydrogen atoms bounded to carbons were located, treated, and refined as riding, considering the isotropic displacement parameter U_iso_(H) = 1.2U_eq_(C) for ternary CH groups [C-H = 0.93 Å], secondary CH_2_ groups [C-H = 0.97 Å], and 1.5U_eq_(C) considered for all methyl CH_3_ groups [C-H = 0.96 Å]. Hydrogen atoms bound to oxygen atoms were located by Fourier maps and by a riding procedure and refined with bond length distances of O-H = 0.82 Å.

### 2.3. X-Ray Powder Diffraction

Powder X-ray diffraction data were acquired using a Bruker D8 Advance diffractometer equipped with a Cu tube CuKa1 (λ = 1.54056 Å), which operates at 40 KV and 40 mA, a Ge (111) monochromator in the incident beam to obtain only CuKa1 radiation, and a LYNXEYE detector. Data acquisition was completed at a scan rate of 0.02°/s.

### 2.4. Differential Thermal Analysis and Thermogravimetric Analysis

Differential thermal analysis (DTA) and thermogravimetric analysis (TGA) were conducted concurrently using a Shimadzu DTG-60H instrument. The samples were placed in an open alumina pan and heated from room temperature to 500 °C at a rate of 10 °C per minute, under a nitrogen gas flow of 70 mL/min. Alumina powder served as the reference sample, and the data analysis was performed using TA60WS software (version 2.20, Shimadzu Corporation, Kyoto, Japan).

### 2.5. Fourier-Transform Infrared Spectroscopy

FT-IR spectra of the samples were obtained using a Jasco 6200 FT-IR spectrometer (Jasco Corporation, Ishikawa-machi, Hachioji-shi, Tokyo, Japan), with 256 scans and 4 cm^−1^ resolution, in the spectral range 4000–400 cm^−1^. The samples were pre-prepared in the form of KBr disks by the pellet technique.

### 2.6. Crystal Energies and Intermolecular Interaction Computation

The strength (magnitude) of intermolecular interaction energies and their nature were investigated and calculated using the CrystalExplorer program [26]. The computation was completed for the asymmetric units and for their neighboring molecules located at distances shorter than the sum of the van der Waals radii. The interaction energies are represented by a sum of four individual energy terms: electrostatic energy (E_ele_), polarization (E_pol_), dispersion (E_dis_), and the exchange repulsion term (E_rep_) [27].

The energy terms were calculated using the [B3LYP/6-31H(d,p)] level of theory. The following scale factors were considered based on the B3LYP energy model, namely k_ele_ = 1.057, k_pol_ = 0.740, k_disp_ = 0.871, and k_rep_ = 0.618.

The values of crystal lattice energies were evaluated using the CrystalExplorer program [26] considering the contributions over a cluster with a radius of 37 Å around the asymmetric units.

The normalized bond lengths were used as follows: CH, CH_2_, and CH_3_ groups (C-H = 1.083 Å) and O-H distances equal to 0.983 Å [28].

### 2.7. Stability

The stability assessment was conducted using the Memmert Humidity Chamber HCP105, which allows the precise control of both relative humidity (having an accuracy of ±1% RH) and temperature (having an accuracy of ±0.1 °C). Periodically, the samples taken from the chamber were analyzed by X-ray powder diffraction to monitor any potential structural changes.

### 2.8. UV Measurements

In vitro solubility tests were performed with the Jasco-V-650 Spectrometer (Jasco Corporation, Ishikawa-machi, Hachioji-shi, Tokyo, Japan) using the LV-724 integrating sphere for UV tests on liquids. The dissolution medium of the samples to be analyzed was deionized water (pH = 6). From a quantity of 3 mg of each sample to be investigated, five stock solutions were prepared in water with the excess sample, which was left stirring (100 rpm) at 37 °C ± 1 °C. After 48 h, the solutions were filtered through a 0.45 μm syringe filter. The concentration of the substance from each analyzed stock solution was calculated against the total volume of water added to obtain the supersaturated solutions. UV analysis of liquid samples was performed in the wavelength range of 200–500 nm, and the sample support was quartz cuvettes (path length 1 cm, V~500 μL).

## 3. Results

### 3.1. Crystal Structures Descriptions

The crystallographic and other refinement-related data of the six solid forms investigated by single crystal X-ray diffraction are given in Table 1.

#### 3.1.1. GW-6

**Figure 1:**
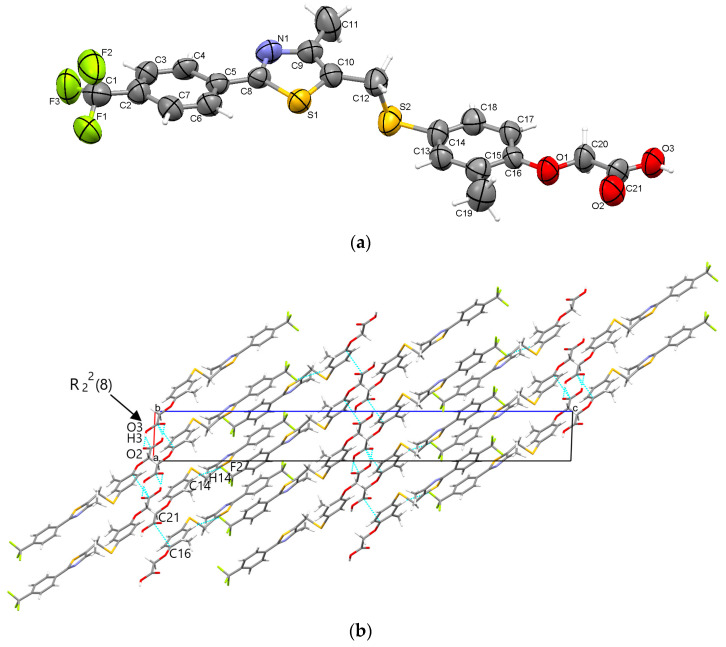
The molecular structure of GW-6 showing the atomic numbering and 50% probability displacement ellipsoids (**a**); a portion of the crystal packing viewed along the b axis and showing intermolecular interactions as thin blue lines (**b**).

The recrystallization of cardarine in methanol yielded the formation of a new polymorph, which was proven to be monoclinic with the centrosymmetric space group P2_1_/n whose asymmetric unit comprises one molecule as depicted in Figure 1a. The crystal exhibits strong mutual carboxyl···carboxyl O3-H3···O2 hydrogen bonds depicting a R^2^_2_(8) homosynthon. The molecule adopts a twisted shape at the methylsulfanyl moiety. The cohesion is also completed by stacking π···π contacts (C21···C16 contact, 3.4 Å) between the carboxyl group and the phenyl ring and C14-H14···F2 interactions of the same phenyl ring with the trifluoromethyl group. The crystal packing diagram observed along the *b*-axis is depicted in Figure 1b.

#### 3.1.2. GW-Bipy

**Figure 2:**
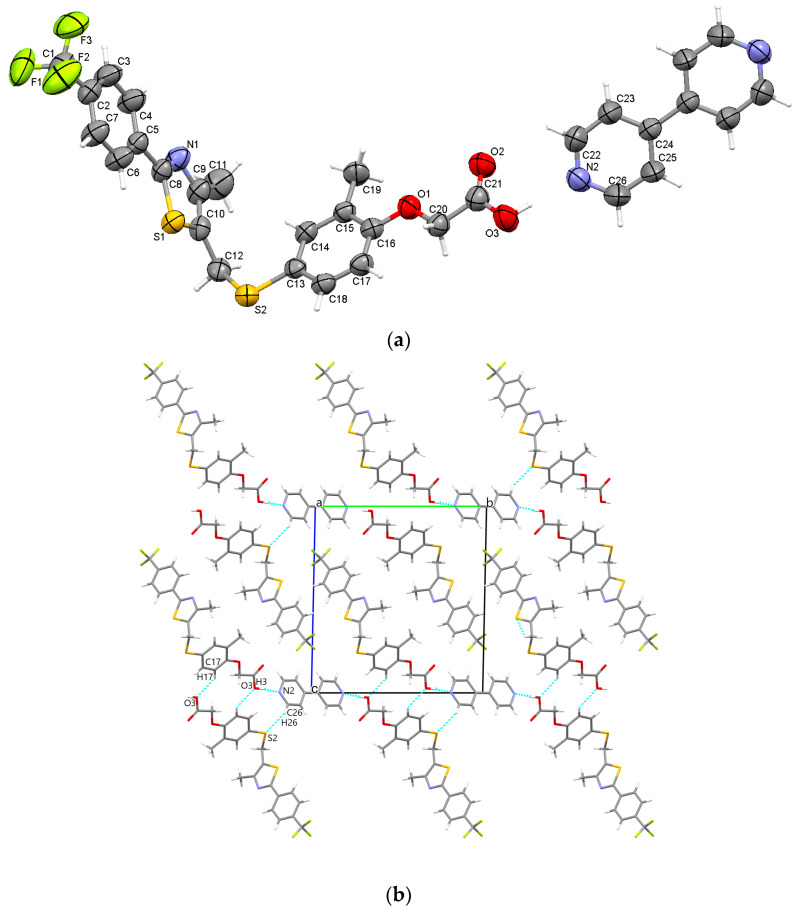
The molecular structure of GW-Bipy 2:1 cocrystal showing the atomic numbering and 50% probability displacement ellipsoids. The bipyridine molecule is situated on inversion center (**a**); a portion of the crystal packing viewed along the a-axis and showing intermolecular interactions as thin blue lines (**b**).

The slow evaporation of the solvent from a mixture of cardarine and 4,4′-bipyridine with various solvents (methanol, ethanol, acetonitrile) led to the self-assembling of a new cocrystal. Using the X-ray diffraction method, it was found to be in a stoichiometric ratio of 2:1 between cardarine to bipyridine and to crystallize in the triclinic crystal system (centrosymmetric P-1 space group) with one cardarine molecule in the asymmetric unit (Figure 2a) and half a molecule of bipirydine, which is located on special positions (namely inversion centers of the unit cell corners). The cardarine molecule is found in a twisted shape in the methylsulfanyl group (Figure 2a). Within the asymmetric unit, the hydroxyl group acts as a donor in the formation of strong hydrogen bridges with the nitrogen of bipiridine. Other intermolecular interactions with distances shorter than the sum of van der Waals radii involved in stability are the C17-H17···O3 interaction between the phenyl ring and carboxyl group and C26-H26···S2 between the phenyl ring and methylsulfanyl moiety. A perspective of crystal packing along the a-axis is illustrated in Figure 2b.

#### 3.1.3. GW-DMF

**Figure 3:**
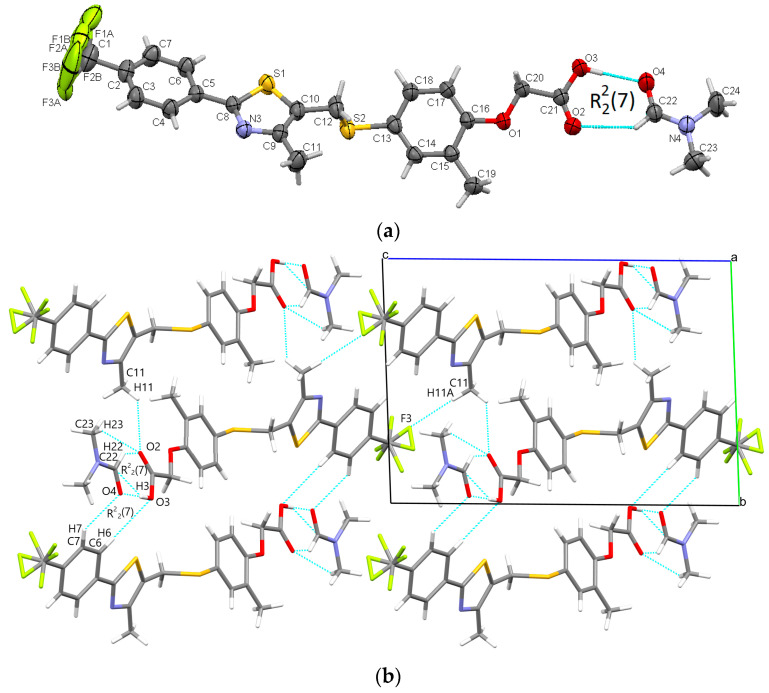
The molecular structure of GW-DMF showing the atomic numbering and 50% probability displacement ellipsoids (**a**); a portion of the crystal packing viewed along the a-axis and showing intermolecular interactions as thin blue lines (**b**).

The recrystallization of cardarine in a solution of dimethylformamide yielded the formation of a solvate, which embedded dimethylformamide molecules within the lattice found in a stoichiometric ratio of cardarine: dimethylformamide of 1:1. The crystal is triclinic centrosymmetric and belongs to the P-1 space group. The asymmetric unit is depicted in Figure 3a and displays a ring like R^2^_2_(7) graph set motif, which links the cardarine and solvent molecule via hydroxyl···carbonyl O3-H···O4 and C22-H···O2 hydrogen bonds. The formation of supramolecular architectures are also sustained by C7-H···O4, C6-H···O3, and O3-H3···O4 hydrogen bonds, which link two cardarine molecules and one solvent molecule in a R^2^_2_(7) motif. The molecular layers of molecules are connected by the solvent molecule, which is linked as well to the C=O carbonyl group of cardarine via the C23-H···O2 bond. The molecular packing in the crystal seen along a-axis is depicted in Figure 3b.

#### 3.1.4. GW-MeOH

**Figure 4:**
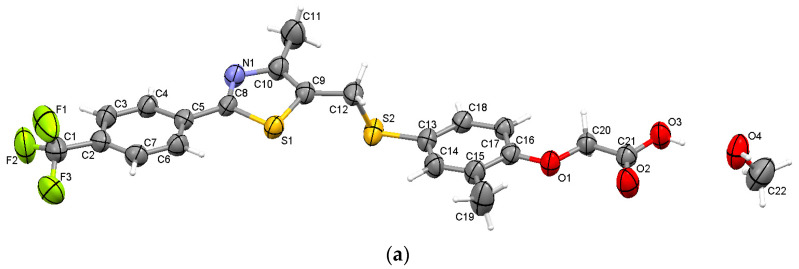

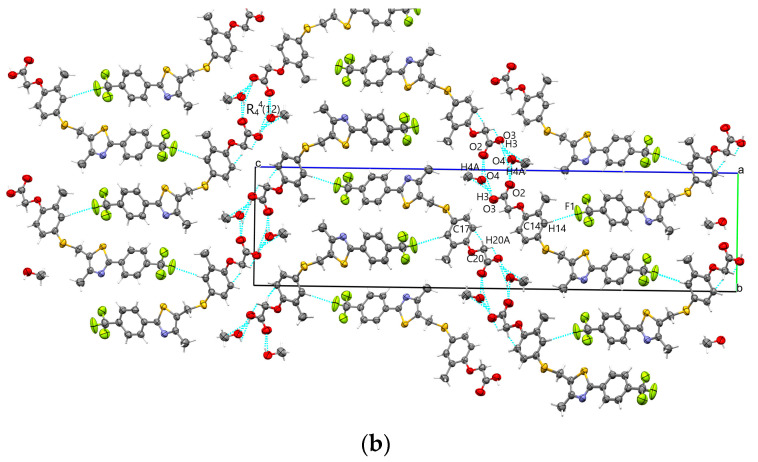
The molecular structure of GW-MeOH showing the atomic numbering and 50% probability displacement ellipsoids (**a**); a portion of the crystal packing viewed along the a-axis and showing intermolecular interactions as thin blue lines (**b**).

The fourth investigated and reported new solid form of cardarine is the methanol solvate, which has formed in a 1:1 stoichiometric ratio as well as its analogous dimethylformamide solvate. The crystal is a monoclinic with P2_1_/c space group with the asymmetric unit being shown in Figure 4a. Two cardarine and two solvent molecules are bridged via O-H···O hydrogen bonds and form a R^4^_4_(12) homosynthon. Along the direction of the a-axis, the molecules are stacked via C-H···π contact (C20-H20A···C17 and C20-H20B···O3) interactions (Figure 4b).

#### 3.1.5. GW-SO

The recrystallization of cardarine in dimethyl sulfoxide yielded to the formation of a new cardarine derivative, which gained an oxygen atom at the S1 sulfur of the methylsulfanyl group; in this way, the valence of S1 is 4 and becomes a sulfoxide group. This transformation involves a thioether (C-S-C) being oxidized to form a sulfoxide (C-SO-C), where one oxygen atom is added to the sulfur. This is a common oxidation process in the pharmaceutical chemistry.

**Figure 5:**
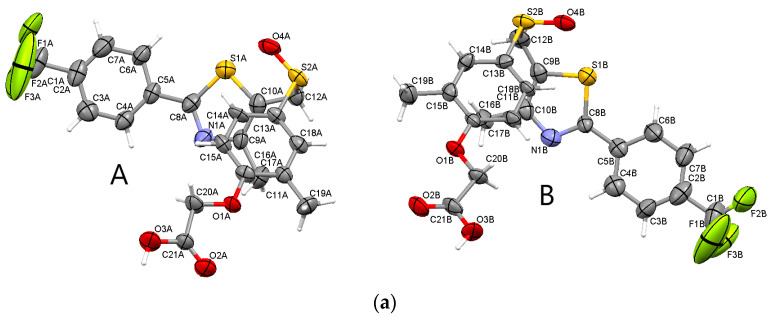

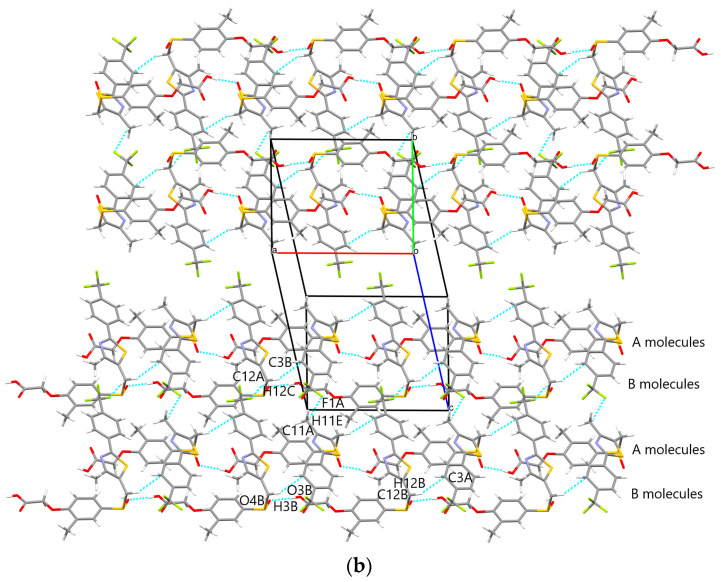
The molecular structure of GW-SO shows the atomic numbering and 50% probability displacement ellipsoids (**a**); a portion of the crystal packing showing intermolecular interactions as thin blue lines (**b**).

The asymmetric unit of the newly crystalline form includes two individual such molecules (denoted by A and B suffixes, Figure 5a). Both molecules depict heavily distorted configurations at the sulfoxide moiety. The layout of supramolecular arrangements is comprised by alternant chains of A and B molecules, which are bridged by carboxyl···sulfoxide (O3B-H3B··O4B and O3A-H3A···O4A) (Figure 5b). Between these molecular chains, C-H···π (C12A-H12C···C3B) and C-H···F interactions (C11A-H11E···F1A and C12B-H12A···F2B) take place.

#### 3.1.6. GW-SO_2_

Another derivative was obtained by recrystallization in a mixture of nitromethane, which is dioxidized at the methylsulfanyl S2 sulfur, thus gaining two oxygen atoms and the valence of sulfur becomes 6. The transformation involves a sulfur atom with two carbons attached (a thioether group, C-S-C) being oxidized to a sulfonyl group (C-SO_2_-C), where two oxygen atoms are added to the sulfur atom. This is a process called oxidation of a thioether to a sulfone (SO_2_) group.

**Figure 6:**
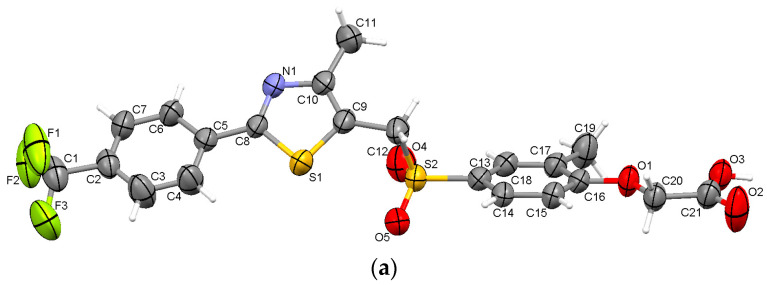

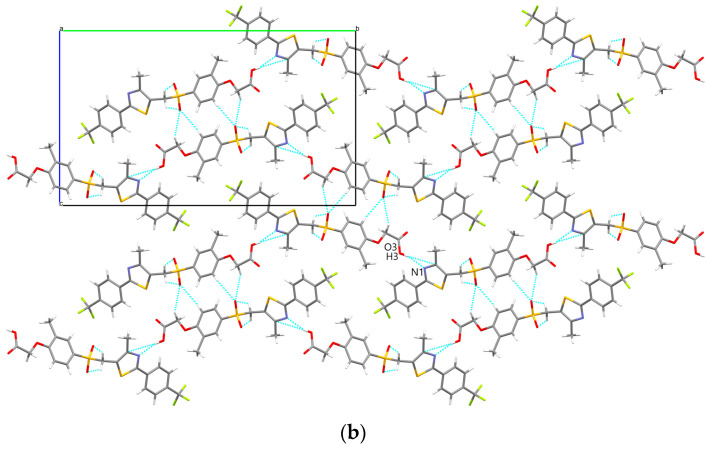
The molecular structure of GW-SO_2_ showing the atomic numbering and 50% probability displacement ellipsoids (**a**); a portion of the crystal packing viewed along the b-axis and showing intermolecular interactions as thin blue lines (**b**).

The asymmetric unit is displayed in Figure 6a, showing the molecule adopting a slightly twisted geometry at the methylsulfanyl group. Unlike in the cases of the other crystals where the thiazole ring was not involved in hydrogen bonding, in dioxidized GW-SO_2_, the carboxyl group is bridged to the nitrogen of the thiazole ring by O3-H3··N1 bonds, which are extended in the direction of the ob-axis. The crystal packing along the ao-axis is depicted in Figure 6b.

The packing and molecular drawings of the studied crystals were generated using Mercury software (Version 2023 3.0) [29]. The intermolecular contacts with distances shorter or equal with the van der Waals radii are listed in Table S1 (Supporting Information).

The conclusions made based on the analysis of the new crystalline forms are as follows:

(i) Cardarine shows a high yield in being prepared in the form of new crystalline forms and derivatives mostly due to the high molecular flexibility exhibited at the methylsulfanyl group (carbon-sulfur C12-S2 bond) due to the involvement of the OH group in the formation of new hydrogen bonds and the embedding of guest molecules;
(ii) All six crystals are centrosymmetric and either monoclinic or triclinic;
(iii) Supramolecular self-assemblies are driven by strong O-H···O and O-H···N hydrogen bonds involving the carboxyl COOH group and the thiazole ring and other van der Waals interactions such as C-H···O, C-H···π, and C-H···F;
(iv) The polymorph exhibits self-assembly synthons of the R^2^_2_(8) type, the GW-DMF solvate has two R^2^_2_(7) synthons, and the GW-MeOH solvate forms an R^4^_4_(12) type synthon.

### 3.2. Powder X-Ray Diffraction Analysis

The X-ray diffraction analysis was conducted with the purpose of checking the structural homogeneity, the purity of the prepared samples, and the proof that the analyzed single crystals are representative of the entire bulk of powder samples. The comparisons of simulated diffraction patterns (which were generated based on the CIF files) and the experimental patterns are illustrated in Figure S1 (Supporting Information). A good match in all paired comparisons can be noticed, with the exception of some diffraction intensities, which are lower in the experimental ones and which account for the preferred orientation of crystallites.

### 3.3. DTA/TGA Thermal Analysis

**Figure 7:**
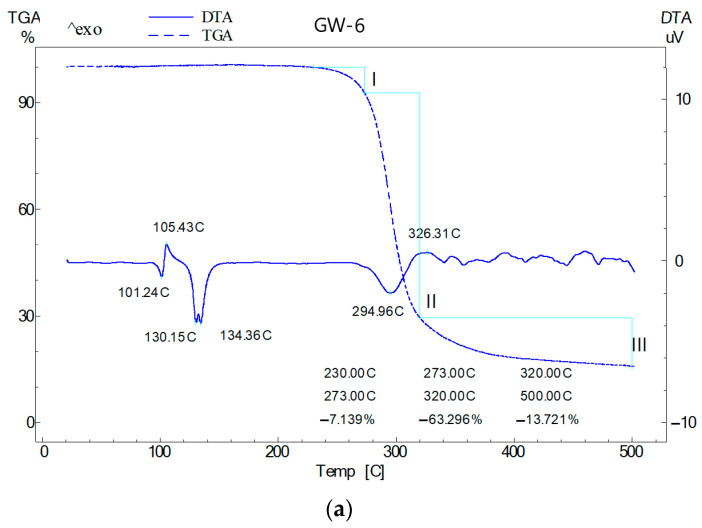

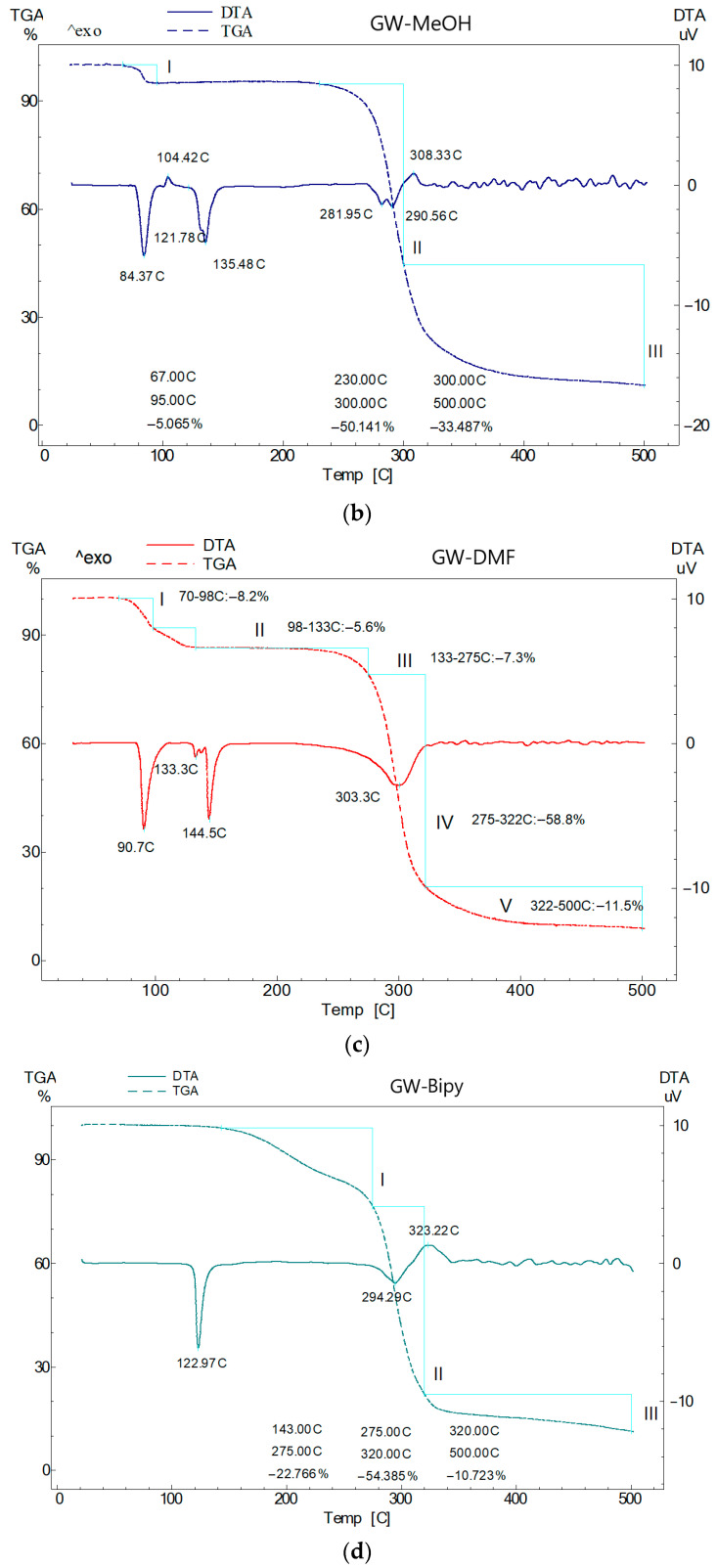

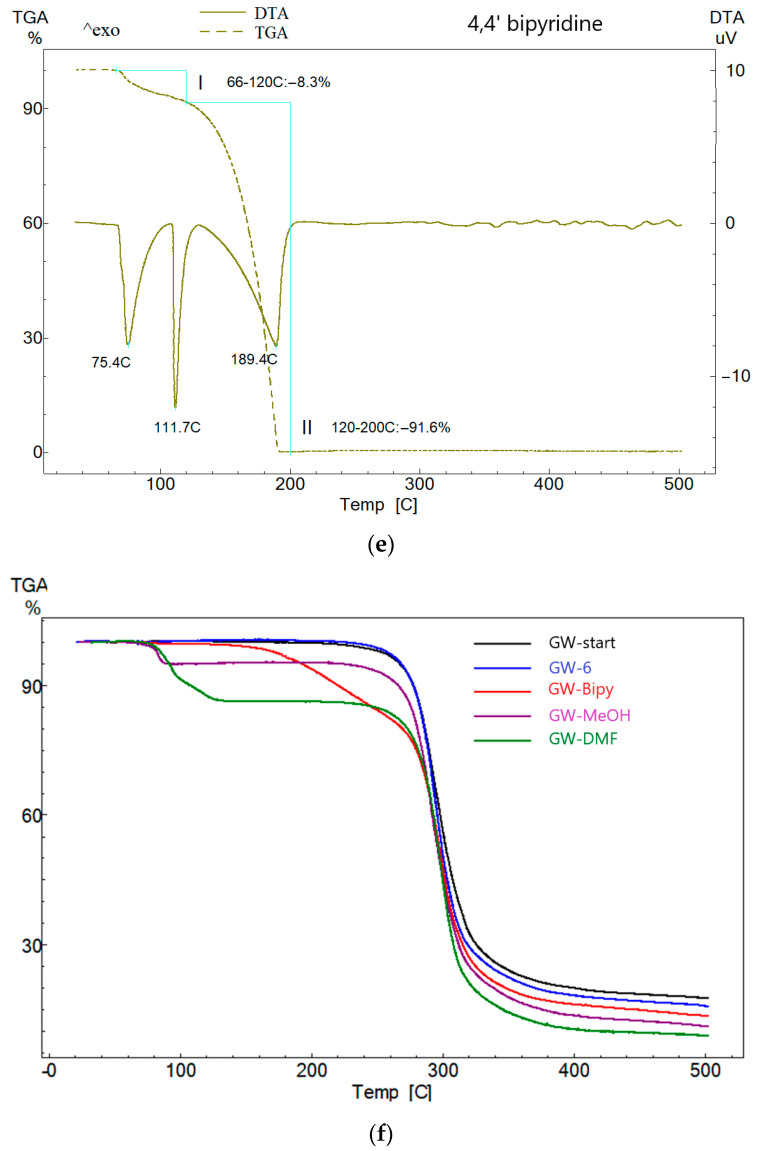
DTA and TGA curves of the analyzed new crystalline forms: the GW-polymorph (**a**); the GW-MeOH solvate (**b**); the GW-DMF solvate (**c**); the 2:1 GW-Bipy cocrystal (**d**); the 4,4′-bipyridine conformer (**e**); and TGA curves of new crystalline forms compared with the GW cardarine start compound (**f**).

Simultaneous DTA/TGA analysis established the thermal characteristics of the new solid forms obtained with cardarine; the curves are illustrated in Figure 7a–f.

For the GW-6 (Figure 7a), an endothermic peak at ~101 °C is assigned to the heat absorbed by the sample during the process of phase transition in a new phase (most likely a new polymorphic phase). Shortly after this event, a small exothermic peak appears at ~105 °C, which can be explained by the crystallization process and the rearrangement of cardarine molecules in the lattice. The two endothermic signals with a maximum at ~130 and 134 °C are attributed to the melting event of cardarine.

The DTA trace of GW-MeOH (Figure 7b) exhibits a sharp endothermic peak at 84 °C, which can be attributed to the loss of methanol molecules found within the crystal lattice, which is accompanied by the TGA graph with an experimental mass loss of 5% due to the loss of methanol molecules, which is quite close to the theoretical solvent content of 6.59%. The slight difference of roughly 1.5% could possibly be due to the fact that not all cardarine molecules within the sample have an associated guest methanol molecule embedded within the lattice; hence, it results in a slightly lower mass loss on the experimental data. At ~104 °C, a small exothermic peak can be noticed, which corresponds to the rearrangement and crystallization of the cardarine molecules, which further undergo the melting process in two steps evidenced by the two endothermic peaks at ~121 °C and 135 °C.

The DTA diagram of the GW-DMF (Figure 7c) solvate manifests a sharp endothermic signal at ~91 °C, which corresponds to the loss of dimethylformamide molecules embedded within the lattice and is accompanied by an experimental mass loss of 8.2% and 5.6%, which accounts for the mass of lost solvent molecules, being exactly the same as the theoretical mass assigned to DMF molecules, which accounts for 13.8% of the total mass. The melting of the cardarine molecules that are left occurs in two steps and is seen as a small endothermic signal at ~133 °C and a sharp protruding endotherm at ~144 °C.

The DTA curve of the cocrystal multicomponent 2:1 ratio of GW-bipy adduct (Figure 7d) shows an endothermic signal with a maximum value at ~123 °C, which is associated with the melting of the multicomponent cardarine-bipyridine system. With increasing heating of the sample, the first weight loss of 21.5% occurs from 140 to 275 °C, which corresponded to the loss of the 4,4′-bipyridine molecules from the melt.

To prove that the cocrystal with 4,4 bipyridine (GW-bipy) has a different thermal behavior compared to the pure coformer (bipyridine), the DTA/TGA curves for it were also recorded (Figure 7d).

The endothermic peak observed in the DTA curve of bipyridine at ~75 °C is attributed to the evaporation of water absorbed on the surface and is accompanied by a mass loss of 8.3%. Furthermore, the sharp peak at ~112 °C is associated with the melting of bipyridine (Figure 7e). Its degradation was evidenced by an endothermic signal with a maximum at ~189 °C, being accompanied by a mass loss of 91.6%. Previous studies have also reported similar thermal behavior for 4,4′-bipyridine [30]. Thus, in the case of pure bipyridine, the mass loss occurs in the temperature range of 111–200 °C, and in the case of the cocrystal, the mass loss due to 4,4′-bipyridine occurs approximately between 130 and 280 °C. Similar thermal analysis was reported in the case of baicalein based cocrystal with 4,4′-bipyridine [31].

From the comparison of the values of the temperature at which the melting of cardarine (Figure 7a) takes place, it can be observed that in the cocrystal (Figure 7c), the event associated with melting occurs at a higher value than in bipyridine (Figure 7d) and lower than in the starting cardarine. This is the consequence of the co-crystal formation between cardarine and 4,4′-bipyridine.

From the comparison of the temperatures at which the thermal events take place, it can be observed that all new solid forms exhibit similar behavior in terms of degradation and oxidation, which occurs at 294 °C for GW-polymorph, 281 °C and 290.5 °C for GW-MeOH, 294 °C for GW-Bipy, and 303 °C for GW-DMF. These processes are also accompanied by a great mass loss of over 50% of the total mass.

The melting process that occurs in successive steps with small temperature differences between the steps (GW-6, GW-MeOH and GW-DMF) can be explained by a partial melting of the compound, followed immediately by a minor structural rearrangement before melting completely.

In Table 2, the values of melting points that are further compared with the ones of the five polymorphs reported previously are summarized (GW-1 to GW-5) [11]. It can be noted that the melting point of the new polymorph (GW-6) is comparable with the other, while the inclusion of guest molecules (GW-MeOH, GW-DMF, and GW-Bipy) led to a slight decrease in the melting points values overall.

### 3.4. Crystal Lattice Energies and Pairwise Intermolecular Energies Evaluation

The lattice energies for the six novel crystals, along with their individual lattice term breakdowns, are presented in Table 3.

With reference to the newly reported polymorph, a total lattice energy of −233.3 kJ/mol is displayed, which is comparable with the other five cardarine polymorphs already reported, which are found between −195.7 kJ/mol and −236.8 kJ/mol [11], which suggests a similar overall stability.

The 2:1 cocrystal with bipyridine shows an overall energy of −151.4 kJ/mol, which is lower compared to the polymorph and indicates a lower stability.

Furthermore, it can be noted that the inclusion of solvent molecules within the lattice lowers the crystal stability, with the computed lattice energies being −152.7 kJ/mol for the methanol solvate (GW-MeOH) and with the dimethylformamide solvate (GW-DMF) being slightly more stable with an energy of –169.9 kJ/mol.

The evaluation of the lattice energies can explain the crystallization of cardarine under the two oxidized forms, thus achieving, from a thermodynamic point of view, new configurations with lower energies compared with the multi-component, especially in the doubly oxidized case (−260.2 kJ/mol in GW-SO_2_), while the oxidized (GW-SO) has a lattice energy of −211.1 kJ/mol only once.

It can be observed that in mono-component crystals, the weight of the electrostatic attraction term plays a more important role in stability among all the attraction terms, representing approximately 33% of the total lattice energy in GW-6, 50% in GW-SO, and 41% in GW-SO_2_. The dominant term in all the structures is by far the dispersion term, which is also reflected by the molecular arrangement in the crystal.

Since we were not able to further reproduce the two oxidized cardarine forms (GW-SO and GW-SO_2_ derivatives) and to evaluate other properties, besides their crystal structures, the conclusion can only be made based on the evaluation of lattice energies. They are more stable compared to the other multi-component solid forms.

The analysis of crystal packing is completed based on the calculation of intermolecular interaction energies with neighboring molecules found at distances shorter or equal than the sum of van der Waals radii. The total interaction energies and the breakdown in four separated terms (E_ele_-electrostatic, E_pol_-polarization, E_dis_-dispersion, E_rep_-repulsion) are given in Table S2 (Supporting Information).

Conclusions emerging from the analysis of energy values are presented below, as follows:

(i) The polymorph is the only structure that displays mutual carboxyl···carboxyl O-H···O hydrogen bonds and is characterized by a higher magnitude of binding energy compared to other crystals mainly due to its electrostatic nature (E_tot_ = −126.2 kJ);
(ii) The interactions between host–guest molecules within the asymmetric units are significant and dominated by the electrostatic terms GW-MeOH (E_tot_ = −85.4 kJ and E_ele_ = −65.9 kJ/mol), GW-DMF (E_tot_ = −110.4 kJ/mol and E_ele_ = −88.3 kJ/mol), and GW-Bipy (E_tot_ = −82.2 kJ/mol and E_ele_ −57.0 kJ/mol);
(iii) The GW-SO_2_ crystal, which is the only displaying hydroxyl···thiazole hydrogen bond, is characterized by a rather high interaction energy (E_tot_ = −79.7 kJ/mol), which is dominated by the electrostatic component as well (E_ele_ = −51.6 kJ/mol);
(iv) In all of the molecular interaction pairs, the polarization energy has the least representative role in cohesion, which indicates that the molecules are not polarized;
(v) A high contribution to the overall stability is given by dispersion energy, which includes the stacking C-H···π interactions.

### 3.5. FT-IR Spectroscopy

By analyzing the compounds through FT-IR spectroscopy, the changes in the vibrational frequencies in the new solid forms obtained were highlighted as a result of the formation of hydrogen bonds, either intra or intermolecular. Figure S2a,b (Supporting Information) showed the infrared spectra for the polymorph, the cocrystal, and the two solvates in comparison.

The spectrum of the multi-component adducts shows a broad absorption band with a peak at 3431 cm^−1^ in GW-MeOH, at 3487 cm^−1^ in GW-DMF, and at 3440 cm^−1^ in the 2:1 GW-Bipy cocrystal due to the stretching vibration of the υ(O-H) bond of carboxyl group present in cardarine molecules. It is interesting to note that the same band is not visible in the GW-6.

The absorption bands in the 3100–2800 cm^−1^ spectral range are due to the stretching vibration of the υ(C-H) bonds in the benzene ring and in the CH_3_ groups as well.

The bands that appear between 2600 and 2500 cm^−1^ correspond to the stretching vibration of the υ(S-H) bond. Even though the S-H group is not present in the molecular composition of cardarine and has not been located by single crystal X-ray diffraction, it is likely that the hydrogen from the carboxyl group COOH migrates to the sulfur S2 and this hydrogen atom is attached to the sulfur in a disordered manner. This explained the lack of the specific O-H stretching in the spectra of the GW-polymorph.

The bands at 1751 cm^−1^ for GW-polymorph, 1780 cm^−1^ for GW-Bipy, 1751 cm^−1^ for GW-MeOH, and 1754 cm^−1^ for GW-DMF are manifested due to the presence of the carbonyl C=O group.

Furthermore, at lower wavenumbers (between 1600 and 1650 cm^−1^), the bands can be explained by the presence of C=C bonds within the six-membered phenyl rings.

Between 1550 and 1400 cm^−1^ in all structures, a low-intensity band associated with the vibration of the stretching C=N group from the thiazole ring can be observed in all spectra.

The bands observed between 1380 and 1000 are due to the vibration of the stretching (C-F) group [32].

In the spectrum of the cocrystal, in addition to the characteristic vibrations from cardarine commented above, a new vibration at 2462 cm^−1^ is observed due to the vibration of the pyridine ring from 4,4′-bipyridine linked by hydrogen bonding to the cardarine molecule. From the comparison of the spectrum of the cocrystal with the spectra of the starting compounds (Figure S4a,b), it was observed that the vibration of the O-H stretching bond from bipyridines appears in the cocrystal at a higher value at 3450 cm^−1^. The wide peak from 2462 cm^−1^ is associated with the stretching (S-H) vibration.

### 3.6. In Vitro Solubility Assessment

A volume of approximately 500 µL of each stock solution was placed in quartz and analyzed in the wavelength range of 200–500 nm (Figure S3, Supplementary Information).

From the superposed of the UV curves, it can be observed that the starting form of cardarine, the new polymorph, cocrystal, and its solvates show an absorbance maximum at a wavelength of approximately 317 nm.

Regarding the absorbance, the highest value recorded is in the spectrum of the cocrystal, followed by the methanol solvate. The UV curve recorded for the cocrystal shows two absorption maxima: the first maximum at 235.5 nm, which is due to the presence of 4,4′-bipyridine, and the second maximum at 317 nm, which is associated with the cardarine signal. These results indicate that after combining with 4,4’-bipyridine molecules, the conformation of cardarine is changed by making hydrogen bonds, leading to an increase in solubility.

Quantitative calibration and analyses: to plot calibration curves, stock solutions of each compound were diluted with water in various concentrations so that the final volume was 1 mL. The values of the obtained concentrations were calculated in mg/mL and are shown in Table S3 (Supplementary Information). The calibration curves are shown comparatively in Figure S3 (Supplementary Information).

Based on the calibration curves obtained for each investigated compound, the concentrations at three dilutions of the stock solution of each compound were calculated. The quantitative analysis was carried out for the same wavelength ʎ = 317 nm.

The calculated values of the concentration of the filtered solutions at which the absorbance is maximum are the following: A_GW_ = 0.81, C_GW_ = 0.070 mg/mL; A_GW-polymprph_ = 0.95, C_GW-6_ = 0.06 mg/mL; A_GW-bipy_ = 1.05, C_GW-bipy_ = 0.072 mg/mL; A_GW-MeOH_ = 1.10, C_GW-MeOH_ = 0.075 mg/mL; A_GW-DMF_ = 0.96, C_GW-DMF_ = 0.079 mg/mL. The difference between the experimental value calculated for the stock solution of the samples and the value obtained using the calibration curve is about 0.005 mg/mL, which represents the mass of the undissolved substance that remained in the syringe filter. From the results of the UV tests, it can be observed that by preparing a new cardarine polymorph, its solubility is slightly lower compared to solvates or the cocrystal.

### 3.7. Stability of the Polymorph and the Cocrystal

The stability of a specific polymorphic form or a cocrystal may be viewed as a disadvantage, as potential phase transitions between polymorphs during extended storage can alter the drug’s physical state, subsequently affecting its shelf life and efficacy. Consequently, it is essential to store the polymorphs in a controlled environment where they are subjected to specific humidity and temperature conditions (75% relative humidity and 40 °C) in order to assess their stability.

The new polymorph and the 2:1 cocrystal samples were stored for up to three months in the climatic chamber and the powder X-ray diffraction patterns were recorded at six-week and three-month intervals, respectively. No significant structural changes occurred in the meantime with the exception that the samples became slightly more amorphous, which indicates that the samples are stable and did not suffer phase transitions (Figure S4, Supporting Information).

## 4. Conclusions

The crystal structures of the four new cardarine-based solid forms and two chemically modified structures were determined and reported. The GW-6, the GW-MeOH, and the GW-SO_2_ forms are centrosymmetric and monoclinic crystals while the GW-bipy, the GW-DMF solvate. and the GW-SO are centrosymmetric triclinic crystals. By the use of the COOH carboxyl group and coupled with the great flexibility at the methylsulfanyl moiety, it was demonstrated that cardarine shows a high potential to be prepared and obtained in the form of new solid forms.

The stability of the crystals is primarily maintained by strong O-H···N hydrogen bonds between the carboxyl and thiazole rings, as well as reciprocal O-H···O hydrogen bonds between carboxyl groups, which make a significant contribution to the electrostatic interaction energy. Another key factor in crystal cohesion is the dispersion interactions, with C-H···π contacts playing the most prominent role in this aspect.

The DTA/TGA analysis has explored the formation of the new multicomponent crystals and assigned their thermal behavior in terms of loss of embedded solvent molecules, the phase transitions that occur for the polymorph, and the methanol solvate before their melting points.

The polymorph and the bipyridine cocrystal are stable and do not undergo phase transitions while the evaluation of their solubility showed a slight improvement for the cocrystal and both solvates compared to the starting form while the solubility of the new polymorph is slightly lower.

## Figures and Tables

**Table 1:** Detailed single crystal X-ray diffraction data of the cardarine-based crystalline forms and derivatives.

**Identification code**	GW-6	GW-Bipy	GW-MeOH	GW-DMF	GW-SO	GW-SO_2_
Empirical formula	C_21_H_18_F_3_NO_3_S_2_	C_26_H_22_F_3_N_2_O_3_S_2_	C_22_H_22_F_3_NO_4_S_2_	C_24_H_25_F_3_N_2_O_4_S_2_	C_21_H_18_F_3_NO_4_S_2_	C_21_H_18_F_3_NO_5_S_2_
Formula weight	453.48	531.57	485.52	526.58	938.97	485.48
Temperature/K	293(2)	293(2)	293(2)	293(2)	293(2)	250(60)
Crystal system	monoclinic	triclinic	monoclinic	triclinic	triclinic	monoclinic
Space group	P2_1_/n	P-1	P2_1_/c	P-1	P-1	P2_1_/n
a/Å	4.8413(6)	4.8765(5)	4.6702(2)	6.6428(2)	11.7781(9)	4.6735(4)
b/Å	10.7961(15)	15.418(2)	10.9176(4)	11.6468(3)	12.8498(14)	27.738(2)
c/Å	40.778(6)	16.734(2)	44.4184(18)	16.5294(4)	15.1481(19)	16.3811(14)
α/°	90	90.787(10)	90	91.443(2)	108.143(10)	90
β/°	92.100(11)	93.949(10)	91.048(4)	96.521(2)	101.939(9)	91.101(7)
γ/°	90	95.890(10)	90	98.027(2)	90.002(8)	90
Volume/Å^3^	2129.9(5)	1248.3(3)	2264.40(16)	1257.02(6)	2126.4(4)	2123.2(3)
Z	4	2	4	2	2	4
ρcalc g/cm^3^	1.414	1.414	1.424	1.391	1.467	1.519
μ/mm^−1^	2.708	2.410	2.617	2.414	0.305	2.831
F(000)	936.0	550.0	1008.0	548.0	968.0	1000.0
Radiation	Cu Kα (λ = 1.54184)	CuKα (λ = 1.54184)	CuKα (λ = 1.54184)	CuKα (λ = 1.54184)	MoKα (λ = 0.71073)	CuKα (λ = 1.54184)
2Θ range/°	6.506 to 143.838	7.748 to 142.094	7.964 to 141.562	7.672 to 141.262	5.698 to 58.392	6.268 to 155.142
Index ranges	−3 ≤ h ≤ 5, −13 ≤ k ≤ 12, −49 ≤ l ≤ 49	−5 ≤ h ≤ 4, −18 ≤ k ≤ 18, −20 ≤ l ≤ 18	−5 ≤ h ≤ 5, −13 ≤ k ≤ 12, −36 ≤ l ≤ 54	−7 ≤ h ≤ 7, −14 ≤ k ≤ 14, −20 ≤ l ≤ 20	−15 ≤ h ≤ 15, −16 ≤ k ≤ 16, 0 ≤ l ≤ 20	−5 ≤ h ≤ 4, −33 ≤ k ≤ 33, −19 ≤ l ≤ 19
Reflections collected	12,724	8016	13,932	24,502	9745	29,479
Independent reflections	4059 [R_int_ = 0.1228, R_sigma_ = 0.1257]	4663 [R_int_ = 0.0324, R_sigma_ = 0.0496]	4318 [R_int_ = 0.0498, R_sigma_ = 0.0420]	4746 [R_int_ = 0.0327, R_sigma_ = 0.0174]	9745 [R_int_ = 0.0942, R_sigma_ = 0.1406]	4062 [R_int_ = 0.1141, R_sigma_ = 0.0818]
Data/restraints/parameters	4059/0/275	4663/1/331	4318/0/294	4746/0/349	9745/0/566	4062/0/292
Goodness-of-fit on F2	1.025	1.027	1.095	1.047	1.044	1.068
Final R indexes [I ≥ 2σ (I)]	R_1_ = 0.1407, wR_2_ = 0.3113	R_1_ = 0.0540, wR_2_ = 0.1330	R_1_ = 0.0646, wR_2_ = 0.1957	R_1_ = 0.0442, wR_2_ = 0.1568	R_1_ = 0.1173, wR_2_ = 0.2813	R_1_ = 0.0774, wR_2_ = 0.2032
Final R indexes [all data]	R_1_ = 0.2255, wR_2_ = 0.3653	R_1_ = 0.0756, wR_2_ = 0.1596	R_1_ = 0.0751, wR_2_ = 0.2059	R_1_ = 0.0468, wR_2_ = 0.1618	R_1_ = 0.2136, wR_2_ = 0.3545	R_1_ = 0.1128, wR_2_ = 0.2332
Largest diff. peak/hole/e Å^−3^	0.46/−0.56	0.23/−0.32	0.27/−0.39	0.18/−0.38	1.16/−0.41	0.43/−0.67

**Table 2:** Melting points of the solid forms compared with the previous reported polymorphs GW1 to GW-5.

Crystal	Melting Point (°C)
GW-1	142
GW-2	137
GW-3	139
GW-4	131
GW-5	139 and 145
GW-6	130 and 134
GW-MeOH	121 and 135
GW-DMF	133 and 144
GW-Bipy	123

**Table 3:** Crystal lattice energies of the six cardarine-based crystalline forms.

Crystal	E_ele_(kJ/mol)	E_pol_ (kJ/mol)	E_disp_ (kJ/mol)	E_rep_ (kJ/mol)	E_latt_ (kJ/mol)
GW-6	−77.7	−18.2	−188.5	51.1	−233.3
GW-Bipy	−37.7	−10.7	−139.9	36.3	−151.4
GW-MeOH	−47.4	−10.1	−141.0	45.8	−152.7
GW-DMF	−50.9	−12.5	−146.7	40.2	−169.9
GW-SO	−107.6	−20.3	−180.5	97.8	−210.6
GW-SO_2_	−107.7	−14.2	−204.7	66.4	−260.2

## Data Availability

On request via email to alexandru.turza@itim-cj.ro.

## Supplementary Materials

CIF files of the cardarine-based crystals were deposited via the Cambridge Crystallographic Data Centre: 2406160 (GW-6); 2406161(GW-Bipy); 2406162 (GW-MeOH); 2406163 (GW-DMF); 2406164 (GW-SO); and 2406165 (GW-SO_2_). The copies can be obtained free of charge on written application to CCDC, 12 Union Road, Cambridge, CB2 1EZ, UK (fax: +44-1223-336033); on request via e-mail to deposit@ccdc.cam.uk; or by access to http://www.ccdc.cam.ac.uk (accessed on 22 January 2025). The following supporting information can be downloaded at https://www.mdpi.com/article/10.3390/pharmaceutics17020152/s1: Figure S1: Experimental and simulated powder X-ray diffraction patterns for the new cardarine-based solid forms and derivatives: GW-6 (a), GW-Bipy (b), GW-MeOH (c), GW-DMF (d), GW-SO (e), GW-SO_2_ (f); Figure S2: FTIR spectra in the 2000–4000 cm^−1^ range (a) and 400–2000 cm^−1^ (b); Figure S3: UV spectra of the cardarine-based solid forms and calibration curves; Figure S4: Diffraction patterns of the polymorph (a) and 2:1 cocrystal (b) kept in the climatic chamber; Table S1: Intermolecular interactions shorter than the sum of van der Waals radii (Å, ^o^); Table S2: Nature and magnitudes of intermolecular interaction energies for selected intermolecular contacts (kJ/mol); Table S3: The concentrations of the solutions used to determine the calibration curves.
