# Supplementary material for: Novel Solid Forms of Cardarine/GW501516 and Their Characterization by X-Ray Diffraction, Thermal, Computational, FTIR, and UV Analysis
Source: Pharmaceutics. 2025 Jan 23;17(2):152. doi: 10.3390/pharmaceutics17020152 (PMC11859518; doi:10.3390/pharmaceutics17020152)
Supplement: Supplementary file 1 [file pharmaceutics-17-00152-s001.zip › pharmaceutics-3381228-supplementary.pdf]

Supplementary Materials: Novel Solid Forms of Cardarine/GW501516 and Their Characterization by X-Ray Diffraction, Thermal, Computational, FTIR, and UV Analysis

Alexandru Turza, Maria Bosca, Marieta Muresan-Pop, Liviu Mare, Gheorghe Borodi and Violeta Popescu

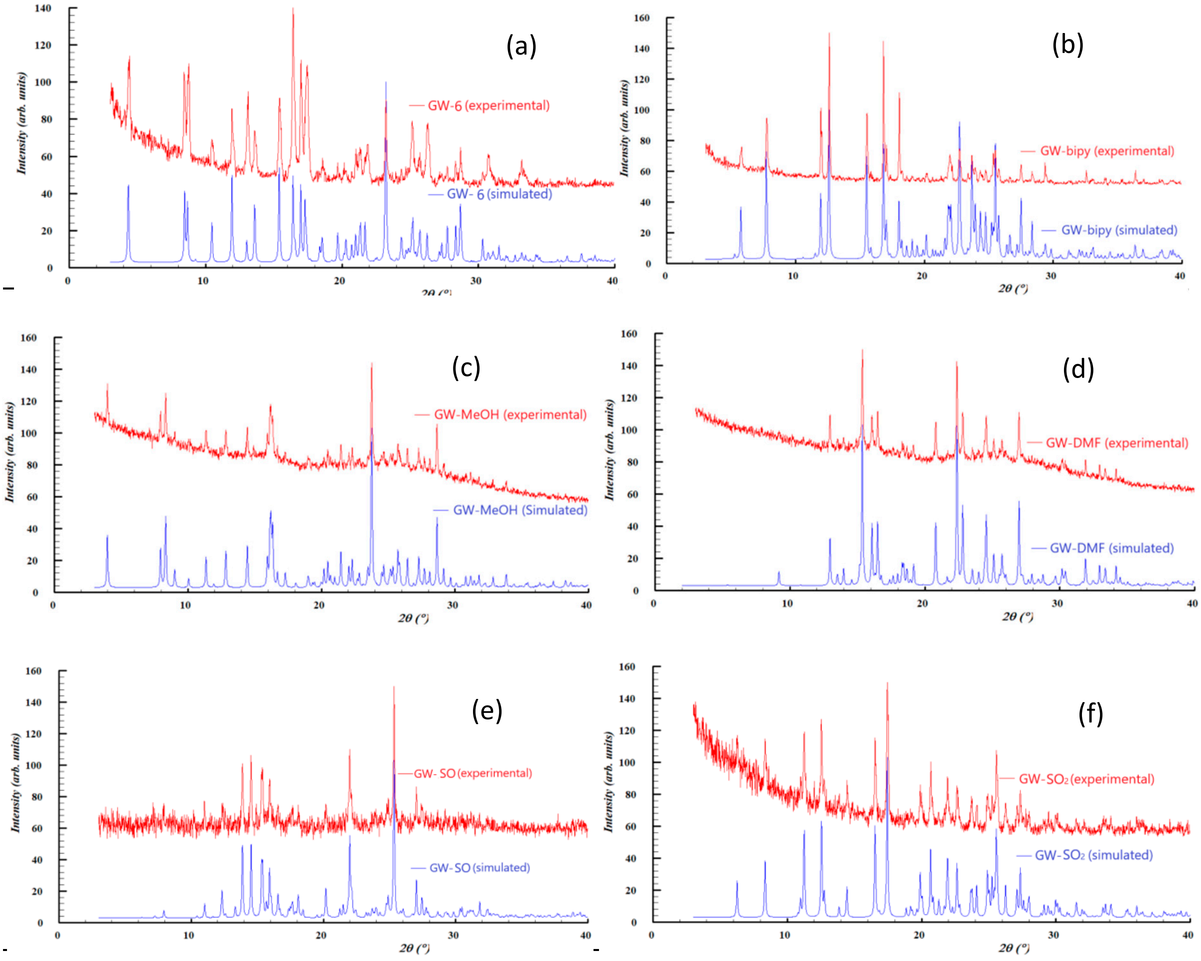

Figure S1. Experimental and simulated powder X-Ray diffraction patterns for the new cardarine based crystalline forms and derivatives: GW-6 (a), GW-Bipy (b), GW-MeOH (c), GW-DMF (d), GW-SO (e), GW-SO_2 (f).

Table S1. Intermolecular interactions shorter than the sum of van der Waals radii (Å, °).

Structure	D-H···A	D-H	H···A	D···A	<(D-H···A)	Symm. op.
GW-6	C14-H14···F2	1.089	2.507	3.566	163.95	1.5-x,-1/2+y,1/2-z
	O3-H3···O2	0.993	1.656	2648	176.47	-2-x,-y,1-z
	O3-H3···C21	0.993	2.555	3.488	156.39	-2-x,-y,1-z
	C3-H3◎ ···C7	1.089	2.896	3.795	139.95	2.5-x,1/2+y,1/2-z
	C6-H6···C4	1.089	2.804	3.721	141.81	1.5-x,-1/2+y,1/2-z
	S2···C18			3.500		1+x,y,z
	C20-H20◎ ···C17	1.089	2.850	3.793	144.86	-1+x,y,z
	C16···C21			3.399		1+x,y,z
	C19-H19◎ ···O2	1.089	2.552	3.438	137.87	1+x,y,z
	C17-H17···H17	1.089	2.349	3.364	154.40	-1-x,1-y,1-z

	C23-H23···F3	1.089	2.627	3.662	158.44	-3-x,1-y,1-z
	C25-H25···F3	1.089	2.628	3.648	155.74	-3-x,1-y,1-z
	C3-H3◎ ···H22	1.089	2.383	3.417	157.93	-2-x,1-y,1-z
	C7-H7···H6	1.089	2.289	3.236	144.26	-1-x,2-y,1-z
	C7-H7···C6	1.089	2.858	3.822	147.62	-1-x,2-y,1-z
	C6-H6···H7	1.089	2.289	2.858	110.45	-1-x,2-y,1-z
	C12-H12B···S1	1.089	2.907	3.790	138.25	1+x,y,z
GW-Bipy	C12-H12◎ ···C14	1.089	2.842	3.739	139.69	1+x,y,z
	C26-H26···S2	1.089	2.821	3.843	156.21	-1-x,1-y,-z
	C19-H19C···O2	1.089	2.613	3.610	151.81	1+x,y,z
	C22-H22···H3◎	1.089	2.383	3.308	141.70	-2-x,1-y,1-z
	C22-H22···O2	1.089	2.713	3.232	108.73	-1+x,y,z
	O3-H3···C22	0.933	2.475	3.382	151.69	x,y,z
	O3-H3···N2	0.993	1.647	2.630	169.77	x,y,z
	O3-H3···C26	0.993	2.698	3.617	154.00	x,y,z
	C17-H17···O3	1.089	2.520	3.422	139.54	-2-x,1-y,-z
	C1-F1···H18	1.307	2.478	3.623	144.62	1/2+x,1/2-y,1/2+z
	C7-H7···H19◎	1.089	2.250	3153	138.83	1.5-x,-1/2+y,1/2-z
	C4-H4···H11◎	1.089	2.138	2.829	118.82	1/2+x,1/2-y,1/2+z
	C11-H11◎ ···C4	1.089	2.829	3.782	146.17	-1/2+x,1/2-y,-1/2+z
	C7-H7···H19◎	1.089	2.250	3.153	138.83	1.5-x,-1/2+y,1/2-z
	C6-H6···O3	1.089	2.716	3594	137.41	1/2-x,-1/2+y,1/2-z
	C6-H6···H3	1.089	2.315	3.132	130.35	1/2-x,-1/2+y1/2-z
	O3-H3···C11	0.993	2.852	3.492	122.87	1/2-x,1/2+y,1/2-z
	O3-H3···N1	0.993	1.801	2.754	159.74	1/2-x,1/2+y,1/2-z
	O3-H3···C10	0.993	2.667	3.547	147.73	1/2-x,1/2+y,1/2-z
GW-SO_2	O3-H3···C8	0.993	2.878	3.752	147.21	1/2-x,1/2+y,1/2-z
	O3-H3···H6	0.993	2.315	2.716	102.95	1/2-x,1/2+y,1/2-z
	C12-H12B···O4	1.089	2.256	3.157	138.66	-1+x,y,z
	C12-H12◎ ···O5	1.089	2.591	3.339	125.15	-1+x,y,z
	C14-O5			3.137		-1+x,y,z
	C14-H14···C14	1.089	2.864	3.448	113.62	1-x,1-y,1-z
	C15-H15···O5	1.089	2.456	3.390	143.15	1-x,1-y,1-z
	C18-H18···F1	1.089	2.478	3.535	163.45	-1/2+x,1/2-y,-1/2+z
	C19-H19◎ ···H7	1.089	2.250	2.990	123.28	1.5-x,1/2+y,1/2-z
	C20-H20◎ ···O5	1.089	2.563	3.441	137.07	1-x,1-y,1-z
	C20-H20B···C16	1.089	2.875	3.753	137.73	-1+x,y,z
	C14-H14···F1	1.089	2.487	3.556	166.60	2-x,1/2+y,1.5-z
	C3-H3◎ ···C6	1.089	2.897	3.937	159.77	3-x,-1/2+y,1.5-z
	C6-H6···C4	1.089	2.832	3.742	141.03	2-x,1/2+y,1.5-z
	C18-H18···O4	1.089	2.717	3.768	162.07	-1-x,1-y,1-z
	C17-H17···O3	1.089	2.650	3.422	127.30	-1-x,1-y,1-z
	C20-H20◎ ···C16	1.089	2.850	3.562	122.98	-1+x,y,z
	C20-H20◎ ···C17	1.089	2.679	3.668	150.84	-1+x,y,z
GW-MeOH	C20-H20◎ ···C18	1.089	2.843	3.886	160.52	-1+x,y,z
	C20-H20B···O3	1.089	2.642	3.395	125.71	1+x,y,z
	C22-H22C···O2	1.089	2.659	3.513	134.82	-1-x,2-y,1-z
	O4-H4◎ ···O2	0.993	1.902	2.827	153.86	-2-x,2-y,1-z
	O4-H4◎ ···C21	0.993	2.826	3.784	162.37	-2-x,2-y,1-z
	O3-H3···O4	0.993	1.622	2.567	157.33	x,y,z
	O3-H3···C22	0.993	2.545	3.300	132.74	x,y,z

GW-DMF	C22-F1B			3.117		2+x,y,-1+z
	C5-F2B			3.150		-1+x,y,z
	C7-H7···O4	1.089	2.449	3.365	140.90	1-x,2-y,1-z
	C6-H6···O3	1.089	2.575	3.555	149.25	1-x,2-y,1-z
	C23-H23B···C3	1.089	2.880	3.804	142.79	-2+x,y,1+z
	C11-H11◎ ···F3B	1.089	2.342	3.430	176.55	2-x,1-y,-z
	C11-H11C···O2	1.089	2.604	3.278	119.34	-x,1-y,1-z
	C12-H12B···O3	1.089	2.713	3.795	172.41	-x,2-y,1-z
	C18-H18···H18	1.089	2.315	3.009	119.89	1-x,2-y,1-z
	C17-H17···H20◎	1.089	2.315	3.009	123.19	-x,2-y,1-z
	C20-H20◎ ···H17	1.089	2.392	3.370	146.72	-x,2-y,1-z
	C19-H19◎ ···H23C	1.089	2.353	3.424	167.45	1+x,y,z
	C23-H23◎ ···O2	1.089	2.570	3.436	135.77	-1+x,y,z
	C22-H22···O2	1.089	2.377	3.140	125.70	x,y,z
	C22-O2			3140		x,y,z
	O3-H3···C22	0.993	2.415	3.328	152.63	x,y,z
	O3-H3···O4	0.993	1.590	2.582	175.76	x,y,z
	C23-H23C···H19◎	1.089	2.353	3.151	128.76	-1+x,y,z
GW-SO	C11◎ -H11E···F1◎	1.089	2.495	3.408	140.62	x,1+y,z
	S2◎ -O2◎			3.255		-1+x,y,z
	O3◎ -H3◎ ···S2◎	0.993	2.702	3.592	149.28	1+x,y,z
	O3◎ -H3◎ ···O4◎	0.993	1.642	2.612	164.27	1+x,y,z
	C21◎ -C21◎			3.375		3-x,1-y,2-z
	C19◎ -H19E···O4◎	1.089	2.639	3.429	128.81	2-x,1-y,2-z
	C20◎ -H20C···S1B	1.089	2.955	4.044	187.67	1+x,-1+y,z
	C20◎ -H20C···C6B	1.089	2.895	3.495	114.87	1+x,-1+y,z
	C11◎ -H11D···H6B	1.089	2.196	3.234	158.62	1+x,-1+y,z
	C11◎ -H11E···F1◎	1.089	2.495	3.408	140.68	x,1+y,z
	C12◎ -H12D···F1◎	1.089	2.593	3.681	177.17	x,1+y,z
	O3◎ -H3◎ ···S2◎	0.993	2.702	3.592	149.29	1+x,y,z
	O3◎ -H3◎ ···O4◎	0.993	1.642	2.612	164.27	1+x,y,z
	C6◎ -H6◎ ···H11C	1.089	2.369	2.999	115.20	x,-1+y,z
	C11B-H11C···H6◎	1.089	2.369	3.292	141.57	x,1+y,z
	C12◎ -H12C···C3B	1.089	2.713	3.672	146.63	x,-1+y,z
	C11◎ -H11D···C6B	1.089	2.825	3.914	177.78	1+x,-1+y,z
	O3◎ -O4◎			2.612		1+x,y,z
	C4◎ -H4◎ ···H4◎	1.089	2.124	3.095	147.01	2-x,-y,1-z
	C12B-H12◎ ···F2B	1.089	2.529	3.617	176.18	x,-1+y,z
	C3B-H3B◎ ···F3B	1.089	2.569	3.637	166.69	1-x,3-y,1-z
	C11◎ -H11D···H6B	1.089	2.196	3.234	158.62	1+x,-1+y,z
	C6B-H6B···H11D	1.089	2.196	2.824	114.39	-1+x,1+y,z
	C19B-H19◎ ···O4B	1.089	2.648	3.442	129.23	1-x,2-y,2-z
	O3B-H3B···O4B	0.993	1.617	2.610	178.32	1+x,y,z
	O3B-H3B···S2B	0.993	2.684	3.592	152.18	1+x,y,z
	O2B-S2B			3.244		x,y,z
	C12B-H12B···C3◎	1.089	2.700	3.631	143.25	-1+x,1+y,z

Table S2. Nature and magnitudes of intermolecular interaction energies for selected intermolecular contacts (kJ/mol).

Crystal	Interaction pair	Contact	E_{ele}	E_{pol}	E_{disp}	E_{rep}	E_{tot}
**GW-6**	Cardarine-Cardarine	O3-H3···O2	-97.4	-17.9	-10.9	0	-126.2

	Cardarine-Cardarine	C20-H20B···O3<br>C21 ···C16 (π···π)	-13.9	0	-93.1	34.8	-73.2
	Cardarine-Cardarine	C6-H6◎ ···C7 (C-H···π)<br>C14-H14···F2	-11.2	-1.3	-29.8	12.1	-30.2
	Cardarine-Cardarine	C3-H3◎ ···C7	0.6	-0.4	-13.8	0	-13.7
	Cardarine-Cardarine	C17-H19◎ ···H17-C17	-2.1	-1.1	-15.6	0	-18.8
**GW-MeOH**	Cardarine-Methanol	O3-H3···O4	-65.9	-12.5	-7.0	0	-85.4
	Cardarine-Cardarine	C20-H20B···O3<br>C20-H20◎ ···C17 (C-H···π)<br>C20-H20◎ ···C16 (C-H···π)<br>C20-H20◎ ···C18 (C-H···π)	-15.9	-2.7	-93.9	38.7	-73.8
	Cardarine-Cardarine	C14-H14···F1	-11.2	-1.1	-29.5	12.2	-29.6
	Cardarine-Cardarine	C6-H6···C4 (C-H···π)	-5.8	-0.7	-18.8	0	-25.3
	Cardarine-Cardarine	C3-H3◎ ···C6 (C-H···π)	-2.3	-0.4	-9.8	0	-12.5
	Cardarine-Cardarine	C18-H18···O4	-3.7	-0.6	-8.4	3.2	-9.5
**GW-DMF**	Cardarine-DMF	O3-H3···O4	-88.3	-15.7	-6.4	0	-110.4
	Cardarine-Cardarine	F2···C15	-3.5	-2.4	-61.5	19.0	-48.4
	Cardarine-Cardarine	C6-H6···O3	-6.6	-1.6	-24.9	10.0	-23.1
	Cardarine -DMF	C7-H7 ···O4	-9.5	-2.4	-8.4	5.2	-15.1
	Cardarine-Cardarine	C11-H11◎ ···O2	-7.1	-2.2	-26.9	10.6	-25.6
	Cardarine -DMF	C23-H23B···C3 (C-H···π)	-1.5	-0.5	-13.3	4.0	-11.3
	Cardarine-DMF	C23-H23◎ ···O2	-12.4	-2.6	-24.2	9.9	-29.3
	Cardarine-Cardarine	C12-H12B···O3	-8.3	-1.5	-22.1	0	-31.9
**GW-Bipy**	Cardarine -Bipyridine	O3-H3···N2	-57.0	-15.2	-10.1	0	-82.2
	Cardarine-Cardarine	C19-H19C···O2<br>C12-H12◎ ···C14 (C-H···π)	-17.3	-3.3	-90.3	32.0	-78.9
	Cardarine-Cardarine	C7-H7···H6-C6	-1.2	-0.8	-19.2	9.0	-12.2
	Cardarine -Bipyridine	C23-H23···F3<br>C25-H25···F3	-3.2	-0.5	-8.6	0	-12.0
	Cardarine-Bipyridine	C22-H22 ···O2	-2.8	-1.0	-9.7	0	-13.5
	Cardarine-Cardarine	C17-H17···O3	-1.1	-0.9	-12.7	0	-14.7
	Cardarine-Bipyridine	C26-H26···S2	-2.5	-0.4	-5.2	5.5	-2.6
**GW-SO_2**	Cardarine-Cardarine	O3-H3···N1	-51.6	-11.5	-16.6	0	-79.7
	Cardarine-Cardarine	C12-H12B···O4<br>C12-H12B···O5<br>C20-H20B···C16 (C-H···π)	-32.5	-6.2	-93.9	42.5	-90.1
	Cardarine-Cardarine	C15-H15···O5<br>C20-H20◎ ···O5<br>C11-H11◎ ···H4-C4	-38.4	-7.2	-36.1	23.1	-58.6
	Cardarine-Cardarine	C18-H18···F1<br>C11-H11◎ ···H4-C4	-1.9	-1.1	-17.2	7.4	-12.8
	Cardarine-Cardarine	C19-H19◎ ···H7-C7	-7.5	-0.6	-15.2	0	-23.3
**GW-SO**	Molecule ◎ -Molecule ◎	O3◎ -H3◎ ···O4◎	-79.2	-12.9	-12.5	53.2	-51.4
	Molecule ◎ -Molecule B	C12◎ -H12C···C3B	-13.8	-3.2	-57.6	21.4	-53.2
	Molecule ◎ -Molecule ◎	C11◎ -H11E···F1◎<br>C12◎ -H12D···F1◎	-2.2	-0.7	-15.1	0	-18.0

Molecule ◎ -Molecule ◎	C19◎ -H19E···O4◎	-9.8	-4.6	-46.1	21.5	-39.0
Molecule B-Molecule B	C12B-H12B···S1B<br>C12B-H12◎ ···C13B (C-H···π)<br>C12B-H12◎ ···C18B (C-H···π)	-23.0	-4.3	-92.5	37.9	-82.2
Molecule B-Molecule B	O3B-H3B···O4B	-84.6	-13.3	-12.7	55.8	-54.8
Molecule ◎ -Molecule B	C11◎ -H11D···C6B (C-H···π)<br>C12B-H12B···C3◎ (C-H···π)<br>C20◎ -H20C···C6B (C-H···π)	-12.4	-3.2	-61.4	25.7	-51.3
Molecule B-Molecule B	C3B-H3B◎ ···F3B	-3.3	-0.6	-14.4	0	-18.3
Molecule B-Molecule B	C11B-H12C···H6◎ -C13B<br>C12◎ -H12C···C3B (C-H···π)	-13.8	-3.2	-57.6	21.4	-53.2
Molecule B-Molecule B	C12B-H12◎ ···F2B	-0.1	-0.1	-7.0	0	-7.2

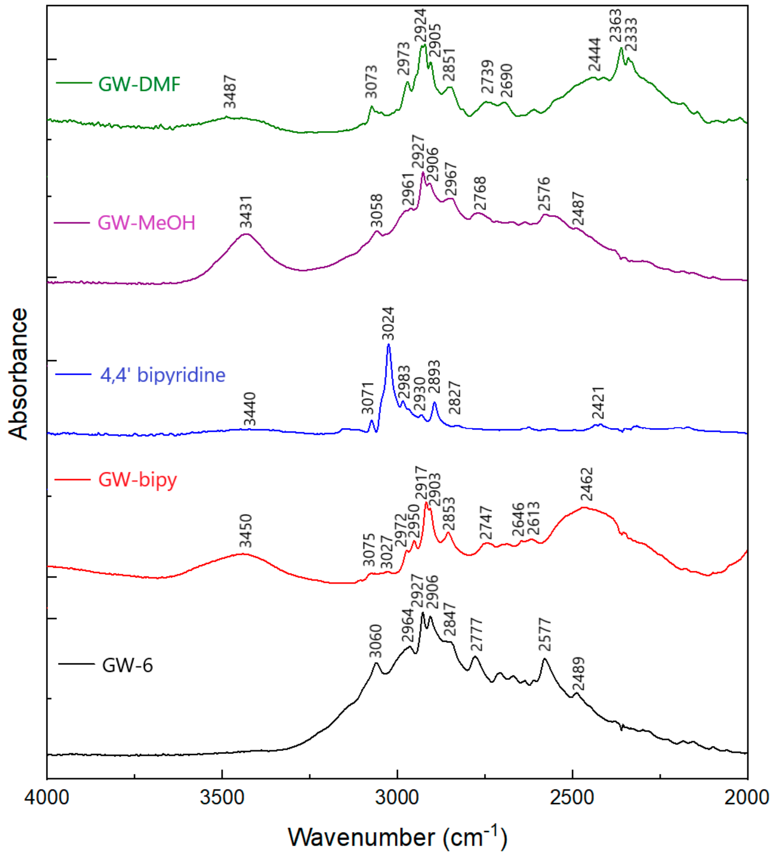

(a)

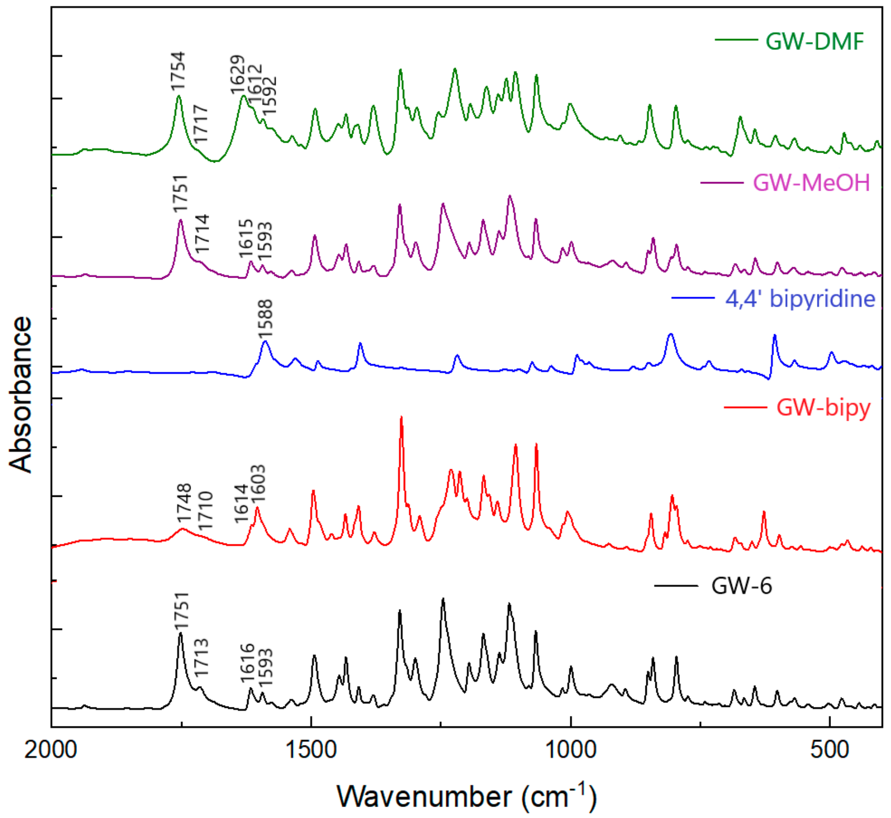

(b)

Figure S2. FTIR spectra in the 4000-2000 cm^{-1} range (a) and 2000-400 cm^{-1} (b).

Table S3. The concentrations of the solutions used to determine the calibration curves.

Volume Compound (mL)	Volume water (mL)	Concentration (mg / mL) / Absorbance 317 nm				
		GW	GW-6	GW-Bipy	GW-MeOH	GW-DMF
**1**	0	0.075 / 0.83	0.065 / 1.01	0.077 / 1.20	0.079 / 1.16	0.084 / 1.01
0.9	0.1	0.0675 / 0.76	0.0585 / 1.11	0.0693 / 0.99	0.0711 / 1.04	0.0756 / 0.90
0.8	0.2	0.06 / 0.70	0.052 / 0.96	0.0616 / 0.90	0.0632 / 0.95	0.067 2 / 0.80
0.7	0.3	0.0525 / 0.61	0.0455 / 0.86	0.0539 / 0.79	0.0553/ 0.81	0.0588 / 0.72
0.6	0.4	0.0450 / 0.52	0.0390 / 0.73	0.0462 / 0.69	0.0474 / 0.71	0.0504 / 0.64
0.5	0.5	0.0375 / 0.43	0.0325 / 0.62	0.0385 / 0.57	0.0395 / 0.59	0.042 / 0.51
0.4	0.6	0.0300 / 0.31	0.0260 / 0.48	0.0308 / 0.48	0.0316 / 0.46	0.0336 / 0.40
0.3	0.7	0.0225 / 0.25	0.0195 / 0.36	0.0231 / 0.35	0.0237 / 0.35	0.0253 / 0.30
0.2	0.8	0.0150 / 0.16	0.013 / 0.23	0.0154 / 0.24	0.0158 / 0.22	0.0168 / 0.19
0.1	0.9	0.0075 / 0.08	0.0065 / 0.16	0.0077 / 0.19	0.0079 / 0.11	0.0084 / 0.10

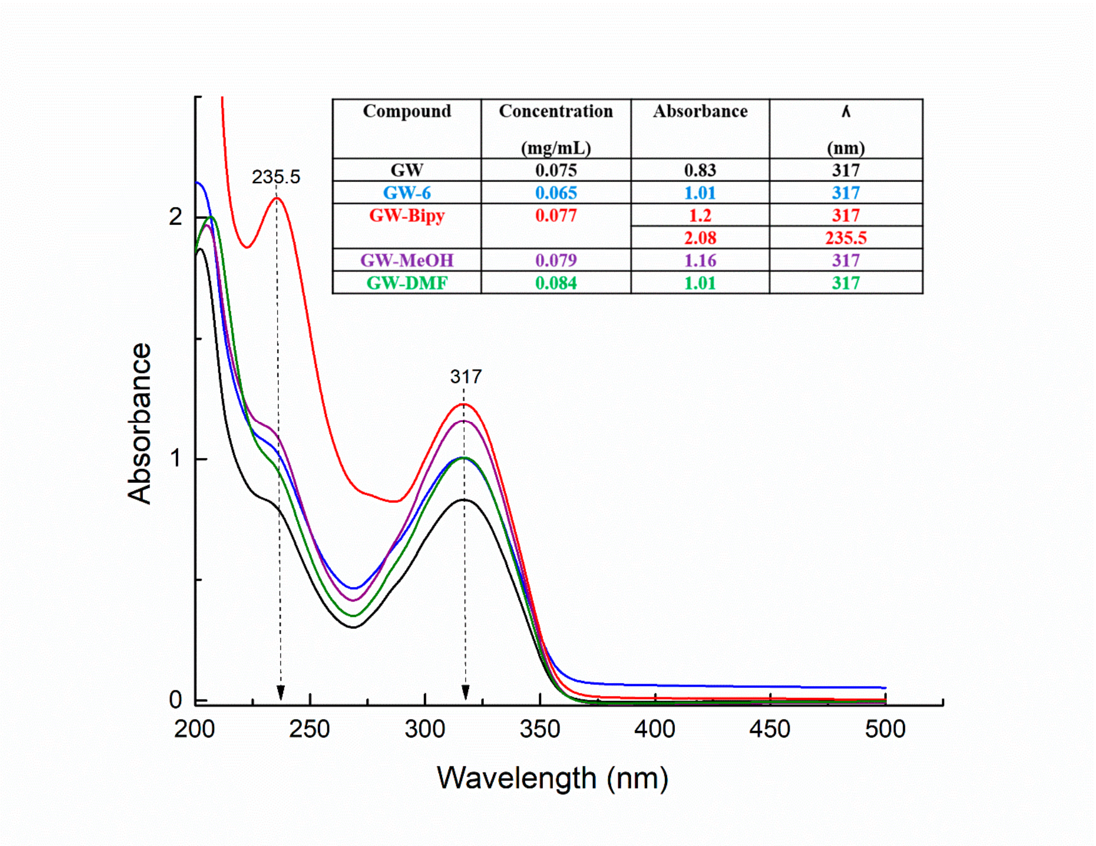
Compound | Concentration (mg/mL) | Absorbance | λ (nm)
GW | 0.075 | 0.83 | 317
GW-6 | 0.065 | 1.01 | 317
GW-Bipy | 0.077 | 1.2 | 317
2.08 | 235.5
GW-MeOH | 0.079 | 1.16 | 317
GW-DMF | 0.084 | 1.01 | 317
235.5
317
Absorbance
Wavelength (nm)
0
1
2
200
250
300
350
400
450
500

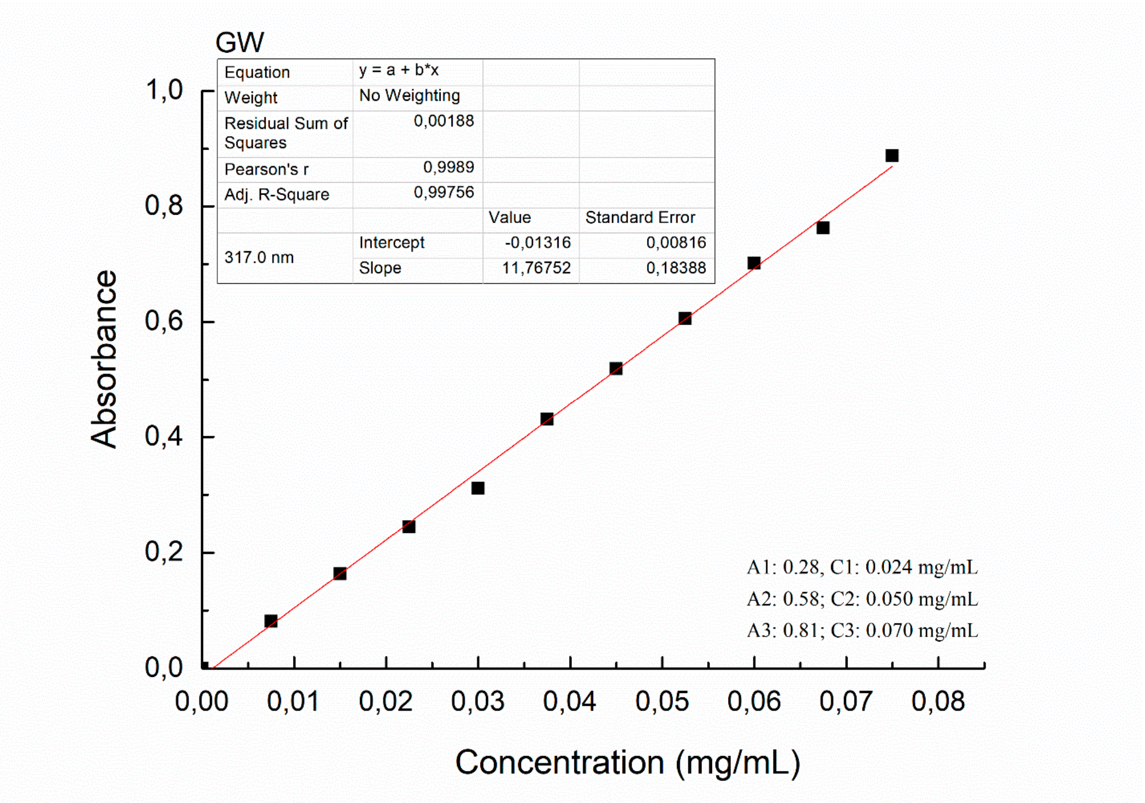
GW
Equation | y = a + b*x
Weight | No Weighting
Residual Sum of Squares | 0,00188
Pearson's r | 0,9989
Adj. R-Square | 0,99756
Value | Standard Error
317.0 nm | Intercept | -0,01316 | 0,00816
Slope | 11,76752 | 0,18388
A1: 0.28, C1: 0.024 mg/mL
A2: 0.58; C2: 0.050 mg/mL
A3: 0.81; C3: 0.070 mg/mL
Absorbance
Concentration (mg/mL)
0,0
0,2
0,4
0,6
0,8
1,0
0,00
0,01
0,02
0,03
0,04
0,05
0,06
0,07
0,08

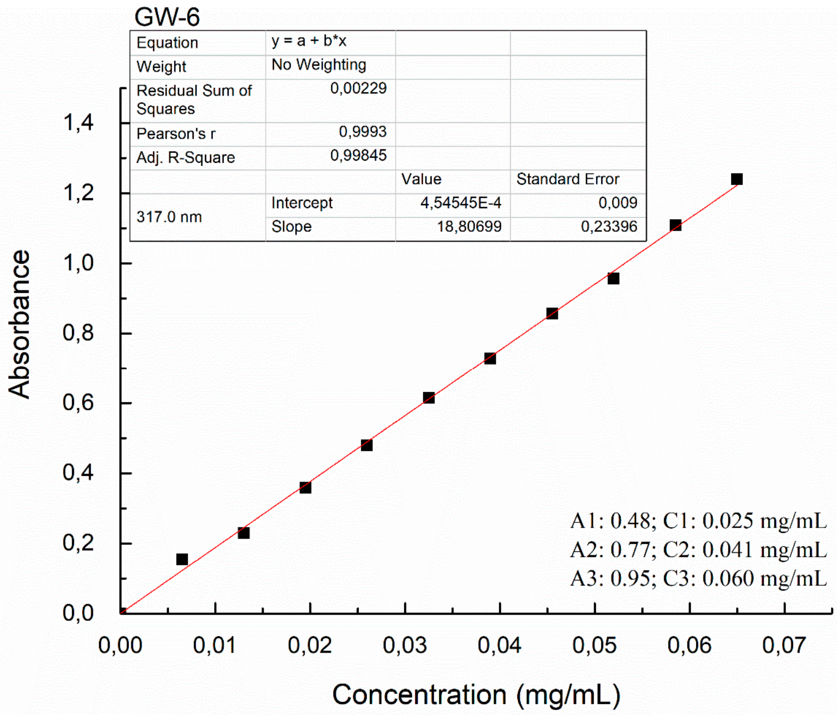
GW-6
Equation y = a + b*x
Weight No Weighting
Residual Sum of Squares 0,00229
Pearson's r 0,9993
Adj. R-Square 0,99845
Value Standard Error
317.0 nm Intercept 4,54545E-4 0,009
Slope 18,80699 0,23396
Absorbance
Concentration (mg/mL)
A1: 0.48; C1: 0.025 mg/mL
A2: 0.77; C2: 0.041 mg/mL
A3: 0.95; C3: 0.060 mg/mL

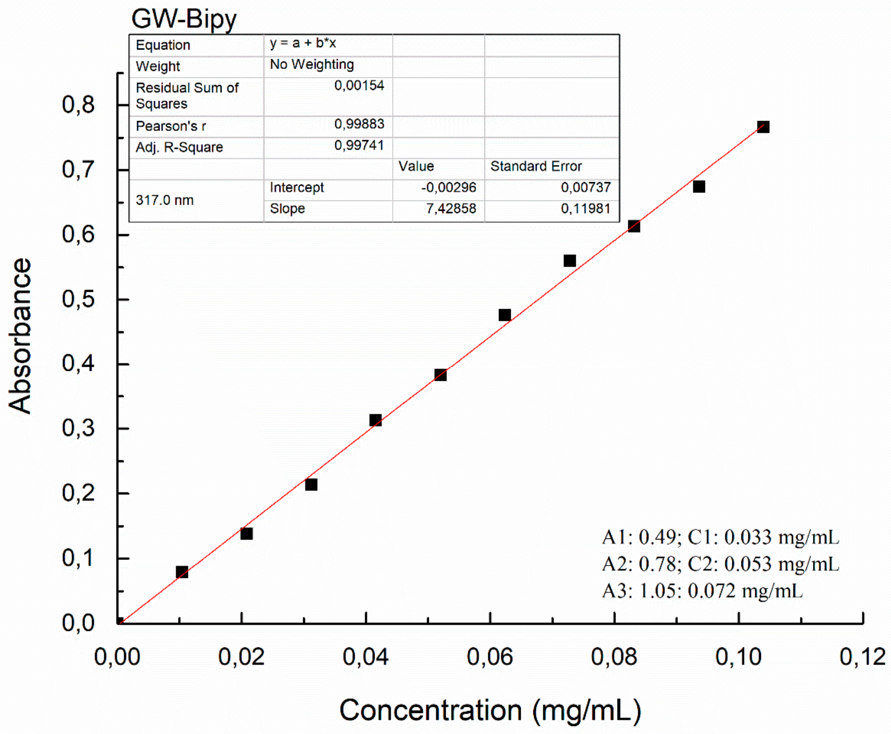
GW-Bipy
Equation y = a + b*x
Weight No Weighting
Residual Sum of Squares 0,00154
Pearson's r 0,99883
Adj. R-Square 0,99741
Value Standard Error
317.0 nm Intercept -0,00296 0,00737
Slope 7,42858 0,11981
Absorbance
Concentration (mg/mL)
A1: 0.49; C1: 0.033 mg/mL
A2: 0.78; C2: 0.053 mg/mL
A3: 1.05: 0.072 mg/mL

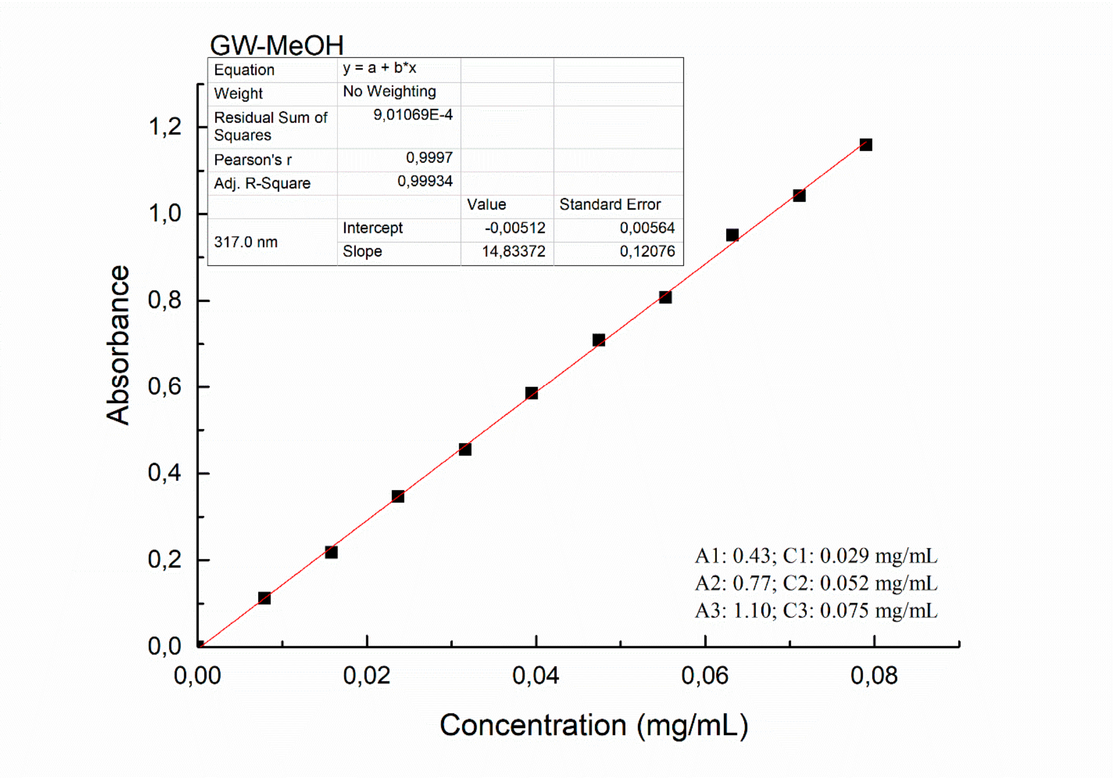

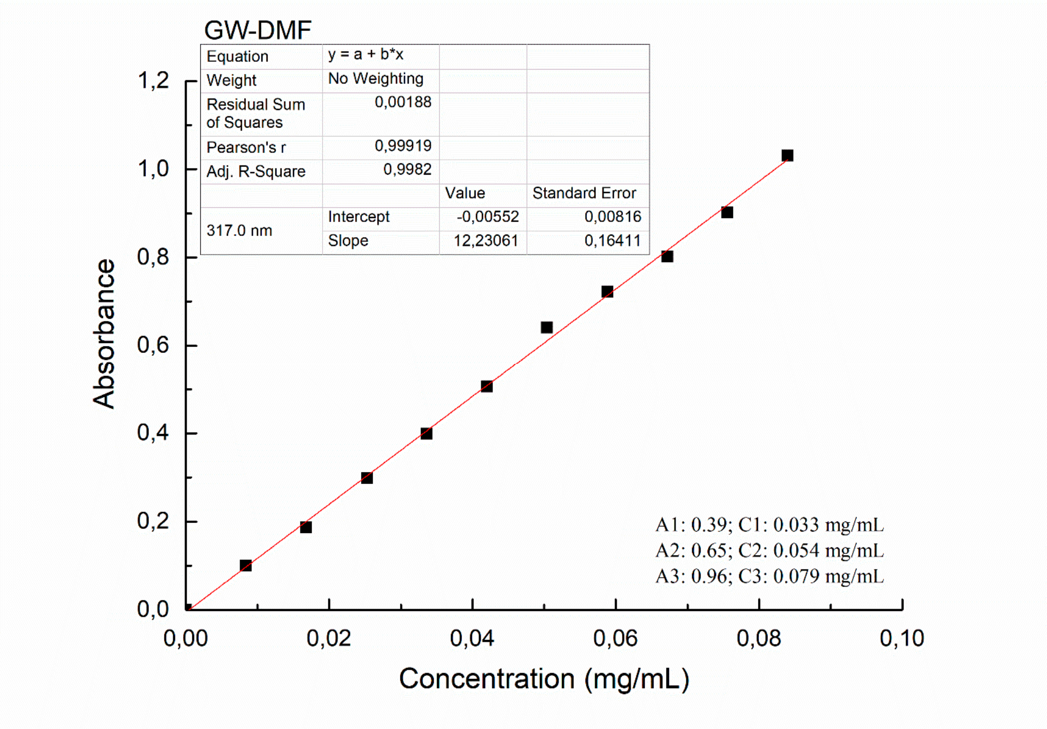

Figure S3. UV spectra of the cardarine based solid forms and calibration curves.

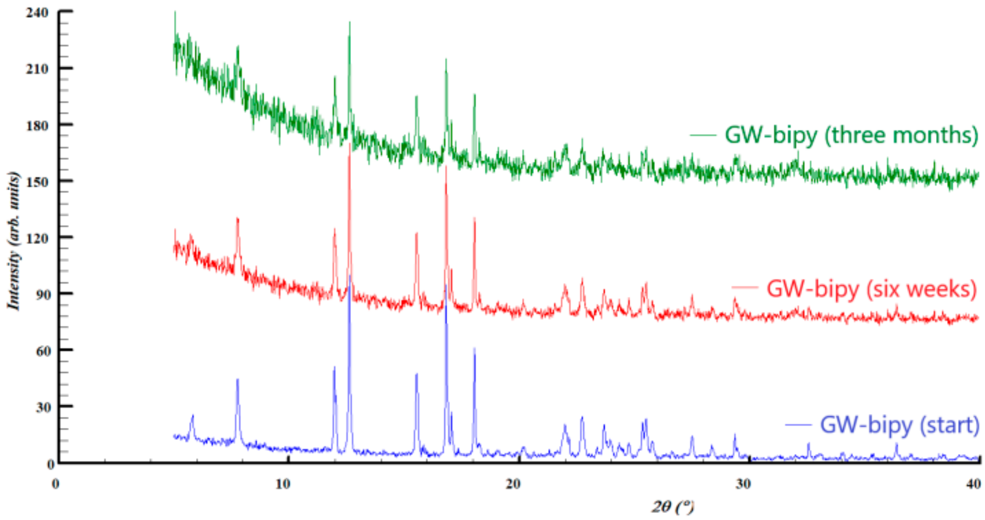

(a)

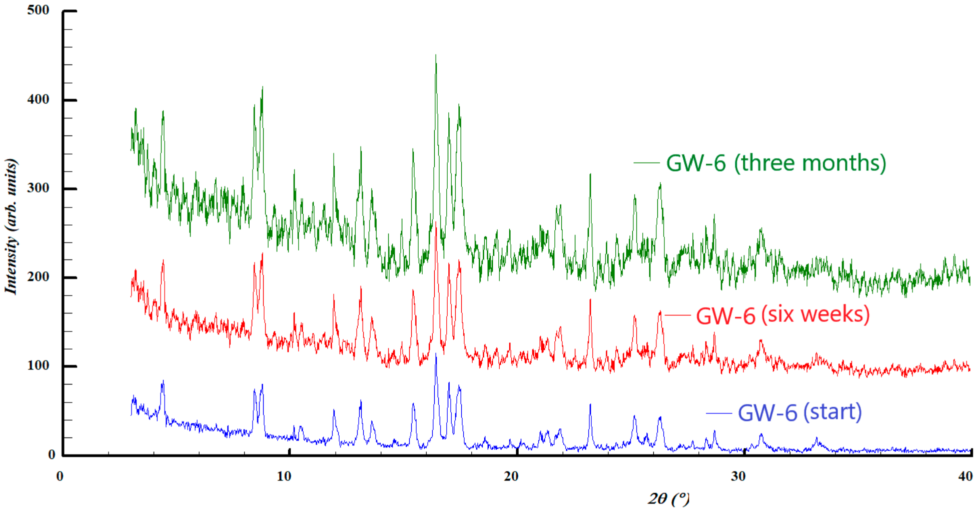

(b)

Figure S4. Diffraction patterns of the polymorph (a) and the 2:1 cocrystal (b) kept in the climatic chamber.
